# A2-type cyclin is required for the asymmetric entry division in rice stomatal development

**DOI:** 10.1093/jxb/ery158

**Published:** 2018-04-26

**Authors:** Xiaoxiao Qu, Min Yan, Junjie Zou, Min Jiang, Kezhen Yang, Jie Le

**Affiliations:** 1 Key Laboratory of Plant Molecular Physiology, CAS Center for Excellence in Molecular Plant Sciences, Institute of Botany, Chinese Academy of Sciences, Beijing, China; 2 University of Chinese Academy of Sciences, Beijing, China

**Keywords:** Cyclin-dependent kinases, cell differentiation, cell division, guard mother cells, cyclin, rice, stomata

## Abstract

In rice, and other major cereal grass crops, stomata are arranged in linear files parallel to the long growth axis of leaves. Each stomatal unit comprises two dumbbell-shaped guard cells flanked by two subsidiary cells. These morphological and developmental characteristics enable grass stomata to respond to environmental changes more efficiently. Cyclin-dependent kinases (CDKs) and their cyclin partners co-ordinate cell proliferation and differentiation during the development of multicellular organisms. In contrast to animals, plants have many more types and members of cyclins. In Arabidopsis, four A2-type cyclins (CYCA2s) function redundantly in regulating CDKB1 activity to promote the asymmetric division for stomatal initiation and the symmetric division of guard mother cells (GMCs). In this study, we examine the function of the single A2-type cyclin in rice, OsCYCA2;1, as well the single B1-type CDK, OsCDKB1;1. Cross-species complementation tests demonstrated that *OsCYCA2;1* and *OsCDKB1;1* could complement the defective stomatal phenotypes of Arabidopsis *cyca2* and *cdkb1* mutants, but also could suppress DNA endoduplication and cell enlargement. The early asymmetric divisions that establish the stomatal lineages are often missing within the stomatal cell files of *OsCYCA2;1*-RNAi rice transgenic lines, leading to a significantly reduced stomatal production. However, GMC divisions are not disrupted either in *OsCYCA2;1*-RNAi or in *OsCDKB1;1*-RNAi rice transgenic lines as expected. Our results demonstrate a conserved but diverged function and behavior of rice A2-type cyclins, which might be associated with the distinct stomatal development pathways between rice and Arabidopsis.

## Teaser

The rice genome contains a single gene *OsCYCA2;1* encoding A2-type cyclin, which is an evolutionarily conserved protein required for asymmetric cell divisions to produce stomatal lineage cells.

## Introduction

Stomata are microscopic valves on aerial surfaces of all land plants regulating the shoot–atmosphere gas exchange. Paleobotanical analyses revealed that stomata originated ~400 million years ago, a key evolutionary innovation formed in the early palaeozoic era (Raven, 2002). Despite the fact that the distribution pattern and morphology are highly diversified in different plants, stomata arise in the epidermis after a series of cell divisions, cell fate changes, and cell shape controls. In the past decades, results of molecular genetic studies demonstrated that stomatal development is an accessible system to reveal the evolution of genes and signals involved in plant development (Vatén and Bergmann, 2012; Ran *et al.*, 2013; Chater *et al.*, 2017; Qu *et al.*, 2017).

In the dicot model plant Arabidopsis, the earliest stomatal precursor, the meristemoid mother cell (MMC), divides asymmetrically (stomatal entry divisions) producing a smaller cell, the meristemoid, as well as a larger sister cell, the stomatal lineage ground cell (SLGC). Meristemoids normally undergo an additional 1–2 rounds of asymmetric divisions (amplifying divisions) to generate new meristemoids before converting into guard mother cells (GMCs). SLGCs can also undergo asymmetric divisions (spacing divisions) to generate satellite meristemoids. Meristemoids differentiate into GMCs after cell fate change. Then, GMCs divide symmetrically to produce paired young guard cells (GCs). During the final stage of stomatal development, GCs undergo cell differentiation, morphogenesis, and pore formation to form functional stomatal units (Bergmann and Sack, 2007).

In contrast to the scattered pattern in dicot Arabidopsis leaves, monocot grass stomata are arranged within linear cell files that parallel the growth axis of the leaf. Stomatal lineage cells initiate at the base of the leaf, and divide asymmetrically to produce two daughter cells, a GMC, and a larger sister cell. At the final stage, GMCs divide symmetrically, producing paired dumbbell-shaped GCs. The stomatal subsidiary cells are produced from cell files flanking the stomatal lineage after asymmetric divisions (Franks and Farquhar, 2007; Liu *et al.*, 2009; Serna, 2011; Raissig *et al.*, 2016).

Cyclins form complexes with specific cyclin-dependent kinases (CDKs) to co-ordinate the cell proliferation and differentiation during the development of multicellular organisms (Swenson *et al.*, 1986; Obaya and Sedivy, 2002). Cyclins, acting as the regulator of CDK activity, contribute to the subcellular localization, substrate specificity, and protein stability of the CDK–cyclin complexes (Dewitte and Murray, 2003; Imai *et al.*, 2006; Boudolf *et al.*, 2009; Boruc *et al.*, 2010). A-type cyclins, known as mitotic cyclins, are essential for the mitotic cell cycle. In contrast to animals, plants encode a large family of A-type cyclins that have been classed into A1, A2, and A3 groups (Vandepoele *et al.*, 2002; Dewitte and Murray, 2003; Wang *et al.*, 2004).

The Arabidopsis genome has four genes encoding A2-type cyclins. *AtCYCA2* genes display tissue- and cell type-specific and overlapping expression patterns, such as in vascular systems and stomatal lineage cells, which are associated with their redundant functions during plant development (Burssens *et al.*, 2000; Imai *et al.*, 2006; Vanneste *et al.*, 2011; Donner and Scarpella, 2013). Mutants of *AtCYCA2* genes frequently form unpaired single guard cells (SGCs), a similar defect of the terminal GMC division to that also observed in mutants of *AtCDKB1* or *AtCDKA;1* genes (Boudolf *et al.*, 2004*b*; Vanneste *et al.*, 2011; Yang *et al.*, 2014). Overexpression of *AtCYCA2:3* as well as *AtCDKA;1* at the late stage of stomatal development induced excessive GC subdivisions (Yang *et al.*, 2014). Arabidopsis *cdkb1;1 1;2* double mutants and *35S:CDKB1;1.N161* dominant negative plants displayed decreased stomatal production and formation of SGCs, indicating that the activity of CDKB1 is required for both meristemoid asymmetric division and GMC symmetric division in stomatal development. In addition to the function in promoting mitosis, AtCYCA2s form functional complexes with CDKs to modulate the cell cycle transition from the mitotic cycle to the endocycle. Genetic suppression of *AtCYCA2* or *AtCDKB1* results in enhanced ploidy levels and enlarged pavement cells (PCs; Vanneste *et al.*, 2011). Co-expression of *CYCA2;3* and *CDKB1;1* induces ectopic cell divisions, limits endoreduplication, and inhibits cell growth (Boudolf *et al.*, 2009).

There are at least 49 putative genes predicted to encode rice cyclins, which were classified into nine types based on evolutionary relationships. Eight of these nine types are common between rice and Arabidopsi*s* (Umeda *et al.*, 1999*a*; Cooper *et al.*, 2003; La *et al.*, 2006). The existence of numerous cyclins implies their diverse regulatory roles in modulating CDK activities during rice development and adaption in response to environmental changes (Cooper *et al.*, 2003; Huang *et al.*, 2008). For example, rice B2-type cyclins, OsCycB2;1 and OsCycB2;2, promote root cell divisions through an association with OsCDKB2;1 (Lee *et al.*, 2003). OsCycH;1 specifically binds to R2 and positively controls CDK and CTD kinase activities to adjust the rate of cell proliferation (Fabian-Marwedel *et al.*, 2002).

In this study, we examine the function of the rice single A2-type cyclin OsCYCA2;1 and OsCDKB1;1 in rice development. Our results demonstrate a requirement for OsCYCA2;1 for stomatal and root development. In contrast to its homolog in Arabidopsis, *OsCYCA2;1* is exclusively required for the asymmetric entry divisions to produce GMCs at the early stage of stomatal development. In addition, combined with phylogenetic analyses, we are providing new clues for further revealing the evolutionary correlation between cell cycle genes and developmental pathways.

## Materials and methods

### Plant materials and growth conditions

The Col-0 ecotype of *Arabidopsis thaliana* L. was used as the wild-type control in the Arabidopsis study. The *cdkb1;1 1;2* double mutants were confirmed by PCR-based approaches (Xie *et al.*, 2010). The *cyca2;34* double mutants were provided by Steffen Vanneste and Tom Beeckman (Vanneste *et al.*, 2011). Seeds were surface sterilized (40 s) in an aqueous solution of 30% (w/v) hydrogen peroxide and 85% (v/v) ethanol in a volume ratio of 1:4 (v/v), and then sown on the surface of half-strength Murashige and Skoog (MS) medium supplemented with 0.8% agar and 1% sucrose. Plants were grown in a controlled temperature and photoperiod chamber at 22 ± 2 °C and 16 h/8 h light/dark illumination cycles. *Oryza sativa* L. spp*. japonica* cultivar Zhonghua 11 was used as the wild-type control and the transformation recipient in the rice study. Rice seeds were soaked in water at 28 °C for 2 d, and then grown in a controlled growth chamber with 30 °C/22 °C day/night temperature cycles, 12 h/12 h light/dark illumination cycles, and 60–70% relative humidity.

### Plasmid construction and generation of transgenic plants

To obtain the construct of gene overexpression, cDNA of *OsCDKB1;1* or *OsCYCA2;1* was cloned into the pH7WG2D.1 vector by using gateway technology and LR Clonase™ II Enzyme Mix (Invitrogen). The recombinant plasmids were confirmed by DNA sequencing before the transformation into Arabidopsis wild type and mutants. To generate RNAi transgenic plants against *OsCDKB1;1* and *OsCYCA2;1*, the conserved sequences from base pair 530 to 695 of *OsCDKB1;1* cDNA and 747 to 979 of *OsCYCA2;1* cDNA were amplified and cloned into pTCK303 vector, respectively. These constructs were electroporated into *Agrobacterium tumefaciens* EHA105 and transformed into rice Zhonghua 11 (Chen *et al.*, 2011). T_1_ seeds were collected to screen positive transgenic plants by using 50 µg l^−1^ hygromycin B (Roche). Real-time quantitative PCR (RT-qPCR) was conducted to confirm the expression level of target genes in transgenic plants. The primer sequences used in this study are listed in Supplementary Table S1 at *JXB* online.

### Real-time quantitative PCR analysis

Rice seedlings were harvested and immediately ground in liquid nitrogen, and the total RNA was isolated using TRNzol reagent (http://www.tiangen.com). The first-strand cDNA was synthesized using a Promega Reverse Transcription kit (http://www.promega.com). RT-qPCRs were performed by using SYBR Premix Ex Taq™ (TaKaRa) with a Corbett RG3000. The *OsACTIN2* gene was used as an internal control. The primer sequences are listed in Supplementary Table S1.

### Yeast two-hybrid assay

The full-length cDNA sequences of *OsCYCA2;1* and *OsCDKB1;1* were amplified using the primers listed in Supplementary Table S1 and cloned into pGBKT7 and pGADT7 vectors (Clontech), respectively. These constructs were transformed into *Saccharomyces cerevisiae* yeast strain AH109 and selected on SD/-Leu-Trp or SD/-Leu-Trp-His-Ade plates. X-Gal activity was then detected.

### Bimolecular fluorescence complementation assay

For bimolecular fluorescence complementation (BiFC) assays, the full-length cDNAs of *OsCYCA2;1* and *OsCDKB1;1* were cloned into pSPYCE-35S and pSPYNE-35S vectors, respectively (Walter *et al.*, 2004). These constructs were transformed into *A. tumefaciens* EH105 and co-injected into tobacco (*Nicotiana benthamiana*). Images were taken after 3 d using a laser scanning confocal microscope (FV1000-MPE, Olympus).

### Pull-down assay

The *OsCYCA2;1* and *OsCDKB1;1* sequences were cloned into pET-28a and pGEX4T-1 vectors, respectively. The *OsCYCA2;1-pET-28a* and *OsCDKB1;1-pGEX4T-1* constructs were transformed into the BL21 strain of *Escherichia coli*. The transformed strains were grown to OD_600_=0.5 under 37 °C and then placed at 18 °C for 30 min. Fusion proteins were induced with 0.4 mM isopropyl-β-d-thiogalactopyranoside (IPTG) at 18 °C for 20 h. The harvested strains (5000 rpm, 10 min, 4 °C) were re-suspended with ice-cold phosphate-buffered saline (PBS) and lysed by sonication. The lysate was centrifuged at 10000 rpm for 60 min and the supernatant was collected. The glutathione *S*-transferase (GST)–OsCDKB1;1 supernatant was loaded on glutathione–Sepharose (GE Healthcare) and washed with PBS. The GST–OsCDKB1;1 fusion protein on glutathione–Sepharose was incubated with the His-OsCYCA2;1 supernatant at 4 °C for 2 h. Then the glutathione–Sepharose was washed with PBS and eluted with 10 mM reduced glutathione elution buffer. The samples were loaded on a 12% SDS–polyacrylamide gel and transferred to a polyvinylidene difluoride (PVDF) membrane (Millipore) by using a semi-dry blotting system (Bio-Rad), and then incubated with anti-His6 monoclonal antibodies followed by horseradish peroxidase (HRP)-conjugated anti-mouse antibodies. The color reaction was performed using the Pro-Light HRP Kit (Tiangen). Signals were exposed to X-ray films and developed.

### DAPI staining and DNA content measurement

Ten-day-old rice roots were fixed in a mixture of 3:1 (v/v) ethanol and acetic acid for 30 min, then rinsed with distilled water. After staining for 30 min with 2 µg ml^−1^ DAPI (Roche) in a staining solution (0.1 M sodium phosphate, 1 mM EDTA, 0.1% Triton X-100, pH 7.0), roots were photographed by a fluorescence microscope. The relative fluorescence intensities were measured using ImageJ software (http://imagej.nih.gov/ij/).

### Flow cytometric analysis

About 20–50 mg of fresh tissue were cut into 2–4 mm fragments and then chopped immediately using a razor blade in 1 ml of Galbraith’s buffer (45 mM MgCl_2_·6H_2_O, 30 mM sodium citrate, 20 mM MOPS, 0.1% Triton X-100, pH 7.0). The cell culture was collected by gentle pipetting and filtered through a cell strainer. The samples were stained with 2 µg ml^−1^ DAPI in an ice bath for 30 min before the analysis using a MoFlo-XDP flow cytometer (Beckman) (Dolezel *et al.*, 2007). A total of ~10000 nuclei were measured for each sample.

### Root semi-thin sections

About 1 cm long primary root tips from 6-day-old rice seedlings were harvested and immersed in cold formaldehyde solution. The samples were subjected to a vacuum for 10 min and placed at 4 °C overnight. The materials were washed with 0.1 M PBS (pH 7.2) four times and were fixed in 1% osmic acid for 1 h, followed by a series of dehydration steps, which were performed by using 30, 50, 70, 80, 90, 100, and 100% ethanol (each step lasted 20 min). Ethanol was substituted with 1:1 acetone and ethanol (v/v) as well as pure acetone twice (each step lasted 20 min). Permeation was performed by using a series of 2:1, 1:1, and 1:2 (v/v) of acetone and epoxy (SPURR) mixture solution. Each step lasted for 3 h. After adding pure epoxy for 8 h, samples were embedded and polymerized at 60 °C for 24 h. Semi-thin sections (thickness 1 µm) were obtained by using a Leica UC7 microtome. Before imaging, sections were stained with 0.1% toluidine blue O.

## Results

### Evolutionary analysis of A2 cyclins in plants

**Fig. 1.**
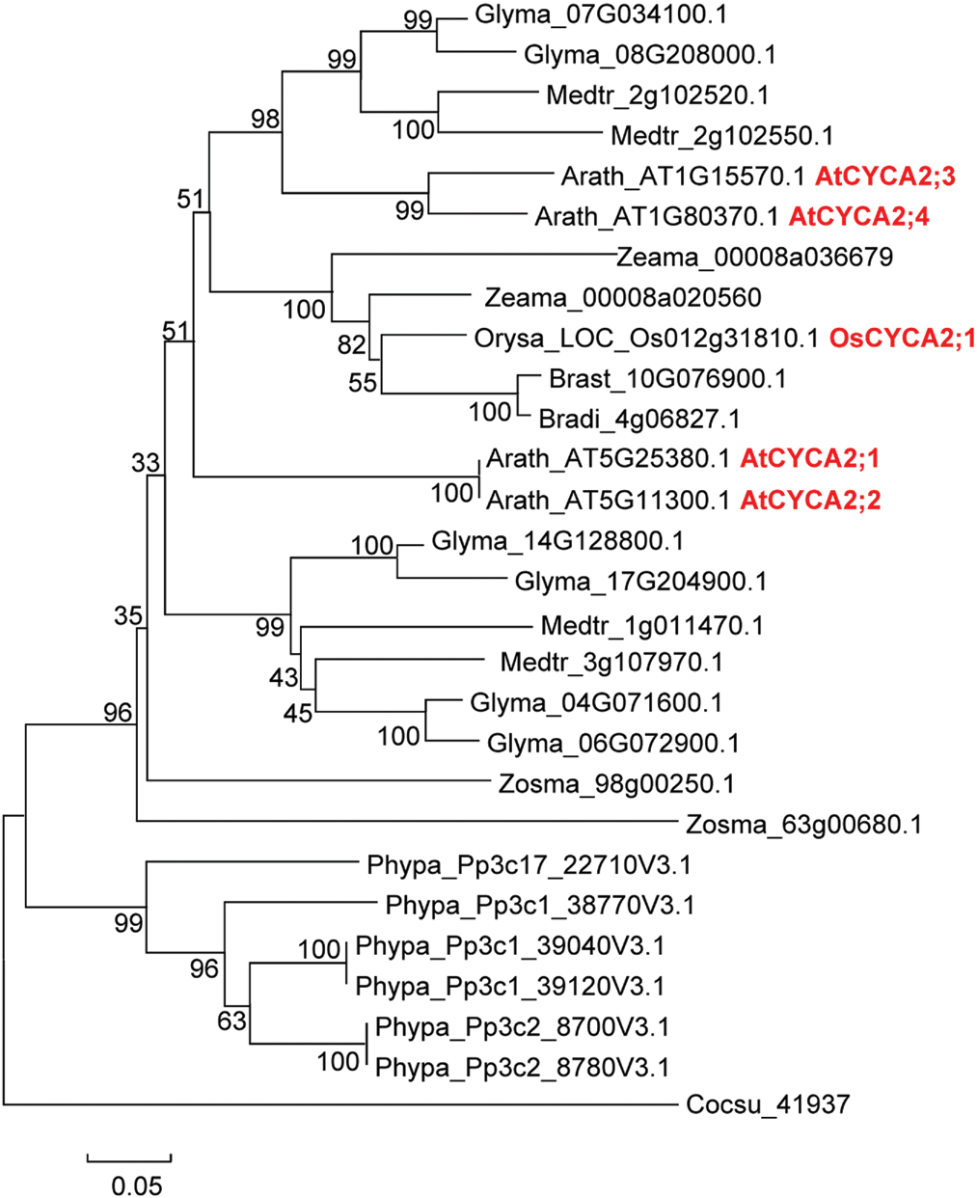
Phylogenetic tree shows that A2-type cyclin-like proteins are conservatively present in green land plants. The phylogenetic tree was constructed using amino acid sequences of Arabidopsis CYCA2 family members based on Phytozome V12.1, using the Neighbor–Joining method in MEGA4. Bootstrap values for 1000 replicates are given in nodes as percentages. Amino acid sequences were used from *Arabidopsis thaliana*, *Brachypodium distachyon*, *Brachypodium stacei*, *Coccomyxa subellipsoidea*, *Glycine max*, *Medicago truncatula*, *Oryza sativa*, *Physcomitrella patens*, *Zea mays*, and *Zostera marina*.

Phylogenetic analysis indicates that homologs of A2-type cyclin are found in lineages that diverged early in the evolution of land plants, before the appearance of stomata. For example, the unicellular green alga *Coccomyxa subellipsoidea* has a *CYCA2* gene in its genome. In the non-vascular land plant moss *Physcomitrella patens*, stomata are exclusively found on the diploid sporophyte (Chater *et al.*, 2016); there are six putative orthologs of CYCA2. In the vascular dicot plants Arabidopsis, soybean (*Glycine max*), and alfalfa (*Medicago truncatula*), the number of *CYCA2* genes was four, six, and four, respectively (Fig. 1). In contrast, the rice genome contains only one copy of the *CYCA2* gene, *Os012g31810*, which is predicted to encode OsCYCA2;1 protein consisting of 490 amino acid residues. Multiple sequence alignment reveals that OsCYCA2;1 shows 40.5% amino acid sequence identity with Arabidopsis CYCA2s, and contains a CDK-binding cyclin box, which is highly conserved among mitotic cyclins (Supplementary Fig. S1) (Umeda *et al.*, 1999*a*). Interestingly, similar to rice, *Brachypodium stacei*, *Brachypodium distachyon*, *Zea mays* (Fig. 1), and many other monocot grasses, *Hordeum vulgare*, *Oropetium thomaeum*, *Panicum hallii*, *Sorghum bicolor*, *Setaria italica*, and *Setaria viridis*, only have 1–2 *CYCA2* genes (Supplementary Fig. S2).

The seagrass *Zostera marina* belongs to basal monocots that returned to the sea. The absence of stomata in *Z. marina* is consistent with the evolutionary loss of entire genes that are required for stomatal development (Olsen *et al.*, 2016). However, like the above grass plants, *Z. marina* possesses two *CYCA2* genes, suggesting that the A2-type cyclin is fundamentally important for plant growth and development, and is not solely linked to stomatal development. The low number of *CYCA2* genes in grasses indicates that *CYCA2* gene duplication might not be necessary, which is associated with their unique developmental pathways and morphogenesis.

### Requirement of *OsCYCA2;1* for stomatal initiation

**Fig. 2.**
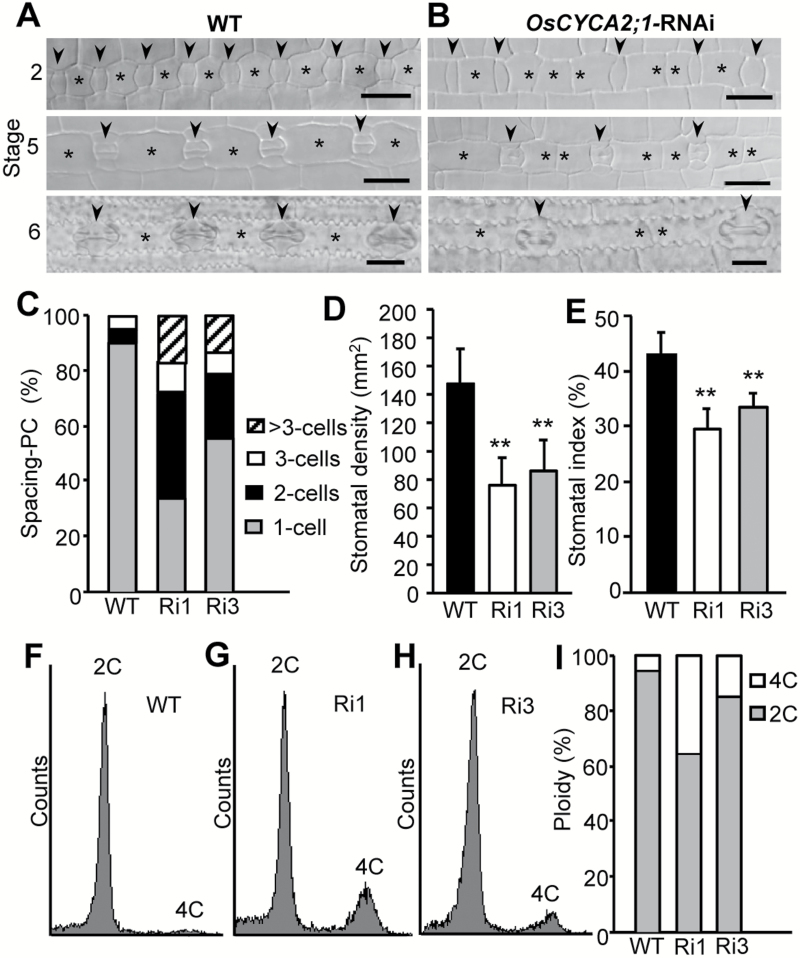
Suppression of *OsCYCA2;1* causes defective cell division in rice. (A, B) Differential interference contrast micrographs of epidermal cells from 6-day-old rice seedlings grown in darkness. Stomata were initiated at the proximal end (base) of young leaves. Asymmetric cell divisions produce a smaller GMC and one larger sister cell (Stage 2, upper panels). The terminal symmetric division of the GMC produces a pair of immature GCs (Stage 5, middle panels). Mature stomatal complexes (a pair of dumbbell-shaped GCs and two flanking SCs) are spaced by one pavement cell (PC) (Stage 6, lower panels). Arrowheads indicate the GMCs or stomatal complexes. Asterisks indicate the PCs that separate stomata in the same cell file. Scale bar=20 µm. (C) The numbers of PCs between two adjacent stomata within the same cell file are often increased in RNAi lines. (D, E) Leaf stomatal density and index of two *OsCYCA2;1*-RNAi lines and the wild type (WT; *n*=12). Data represent the mean ±SD. Asterisks indicate a significant difference from WT controls (Student’s *t*-test, **P<0.01). (F–H) Flow cytometric analysis of nuclei in shoot cells. (I) Quantitative analysis of DAPI fluorescence. *OsCYCA2;1*-RNAi transgenic lines have a higher average 4C DNA content than the WT. For each line, ~10000 cell nuclei were measured.

To elucidate the function of A2-type cyclin in rice development, RNAi transgenic rice lines targeting *OsCYCA2;1* were generated. Transcript levels of *OsCYCA2;1* in two lines, Ri1 and Ri3, were suppressed to 28% and 61%, respectively, in relation to the level in wild-type rice seedlings (Supplementary Fig. S3A). In rice leaf epidermis, stomata form within the stomatal lineage files following a gradual base to tip maturation pattern; the developing stomata can only be found at the proximal end (base) of the leaf. Unlike in Arabidopsis, GMCs in rice are produced directly by asymmetric entry divisions without the precursor stage of the meristemoid. Each undifferentiated cell close to the base of the leaf divides asymmetrically and generates one smaller GMC and one larger sister cell (Stage 2, upper panel of Fig. 2A). Subsidiary mother cells (SMCs) flanking the GMCs are produced by the cells in the neighboring cell files. The terminal division of GMC produces a pair of immature GCs (Stage 5, middle panel of Fig. 2A). Within the wild-type stomatal lineage cell files, stomatal complexes are spaced by one lobbed PC (Stage 6, lower panel of Fig. 2A). However, in *OsCYCA2;1*-RNAi transgenic plants, more than two spacing cells were often observed between two neighboring GMCs/stomata within the same cell file (Fig. 2B, C), leading to a decreased stomatal density and stomatal index (Fig. 2D, E).

Mutations of Arabidopsis *CYCA2* genes caused a failure of GMC division, leading to the formation of aberrant stomatal units (SGCs) (Vanneste *et al.*, 2011). However, the structure and morphology of mature stomata in *OsCYCA2;1*-RNAi rice transgenic plants are indistinguishable from those of wild-type stomata, indicating that the subsequent GMC symmetric divisions as well the subsidiary cell asymmetric divisions are not interrupted by the suppression of *OsCYCA2;1* (lower panels in Fig. 2A and B). Taken together, the above observations indicate that *OsCYCA2;1* is essentially required for the asymmetric entry division during stomatal initiation at the early stage of stomatal development, but not for the terminal GMC symmetric divisions and subsidiary cell asymmetric divisions.

It has been demonstrated that Arabidopsis CYCA2s not only promote cell proliferation but also negatively regulate endocycle onset (Imai *et al.*, 2006; Yoshizumi *et al.*, 2006; Vanneste *et al.*, 2011). Flow cytometric analysis showed that in wild-type rice, only 6% of cells showed a 4C DNA content, whereas most cells were 2C (diploid). However, in *OsCYCA2;1*-RNAi transgenic rice lines Ri1 and Ri3, the fraction of 4C cells markedly increased to 36% and 18%, respectively. Higher ploidy levels, like in Arabidopsis *cyca2* mutants (8C, 16C, 32C), were barely detectable in rice *OsCYCA2;1*-RNAi plants (Fig. 2F–I).

### *OsCYCA2;1* complements epidermal defects of Arabidopsis cyca2 mutants

**Fig. 3.**
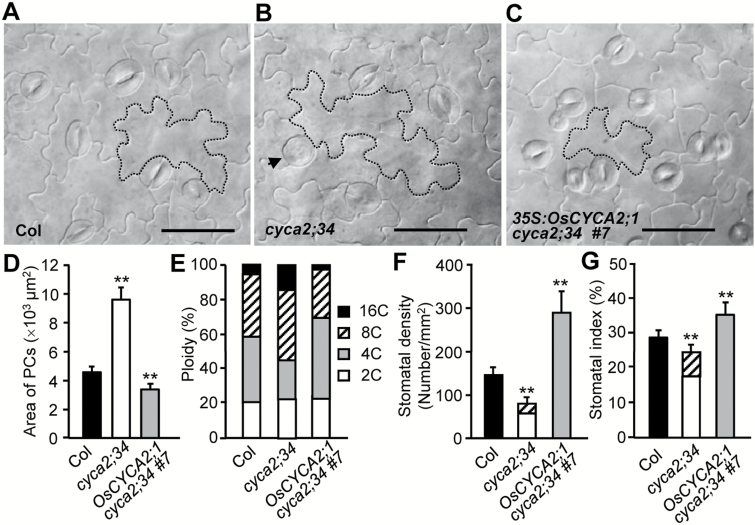
Cross-species expression of *OsCYCA2;1* complements the epidermal defects of Arabidopsis *cyca2;34* mutants. (A–C) Differential interference contrast micrographs of cotyledon epidermal cells of 14-day-old Arabidopsis seedlings of the Col, *cyca2;34*, and *cyca2;34* harboring *35S:OsCYCA2;1*, Line *#7*. An arrow points to a single guard cell (SGC). Representative pavement cells (PCs) are traced with dashed lines. Scale bar=50 µm. (D) Comparison of PC area (*n*=30). (E) Proportions of cells with different ploidies. (F and G) Stomatal density and index. The diagonal line-filled box indicates the SGCs. Data in (D, F, G) represent the mean ±SD. Asterisks indicate a significant difference from Col wild-type controls (Student’s *t*-test, **P<0.01).

The Arabidopsis epidermis is an ideal system to identify gene functions in plant development programs and morphogenetic patterns. To identify further the function of *OsCYCA2;1* in epidermal development, *OsCYCA2;1* coding sequences driven by 35S promoters were transformed into Arabidopsis *cyca2;34* mutants. Compared with the wild type, *cyca2;34* mutants display enlarged pavement cells and enhanced ploidy levels. Cross-species expression of *OsCYCA2;1* (line #7) inhibits the abnormal PC enlargement in *cyca2;34* epidermis, to a cell size even smaller than in the wild type (Fig. 3A–D). Moreover, revealed by flow cytometric analysis, expression of *OsCYCA2;1* is able to inhibit the high DNA ploidy levels in *cyca2;34* mutants (Fig. 3E; Supplementary Fig. S4). This result is consistent with the previous findings that overexpression of *CYCA2* genes could restrain endoreduplication in Arabidopsis (Imai *et al.*, 2006; Boudolf *et al.*, 2009).

In *cyca2;34* mutants, ~10% of GMCs failed to divide symmetrically and formed into SGCs (Fig. 3B, arrow). Strikingly, *OsCYCA2;1* expression can fully rescue the defective GMC division in *cyca2;34* epidermis, suggesting that rice OsCYCA2;1 remains a conserved function in promoting the GMC symmetric divisions. In addition, expression of *OsCYCA2;1* in the *cyca2;34* mutant background induced formation of excessive stomata, reflected by an increased stomatal density and stomatal index (Fig. 3F, G). In another *35S:OsCYCA2;1 cyca2;34* transgenic line (line #8), the relative transcript level of *OsCYCA2;1* is much lower than in line #7; the defective GMC division and reduced stomatal production are partially rescued, indicating that *OsCYCA2;1* quantitatively promotes stomatal development depending on its expression level (Supplementary Fig. S5). Taken together, our results of cross-species complement tests demonstrate the conserved abilities of *OsCYCA2;1* in limiting cell endoreduplication and PC size, as well as in rescuing *cyca2;34* defective asymmetric entry divisions (for stomatal initiation) and symmetric GMC divisions (for guard cell formation), despite *OsCYCA2;1* being functionally required only for stomatal entry divisions in rice.

### OsCYCA2;1 is also required for cell division and differentiation in roots

**Fig. 4.**
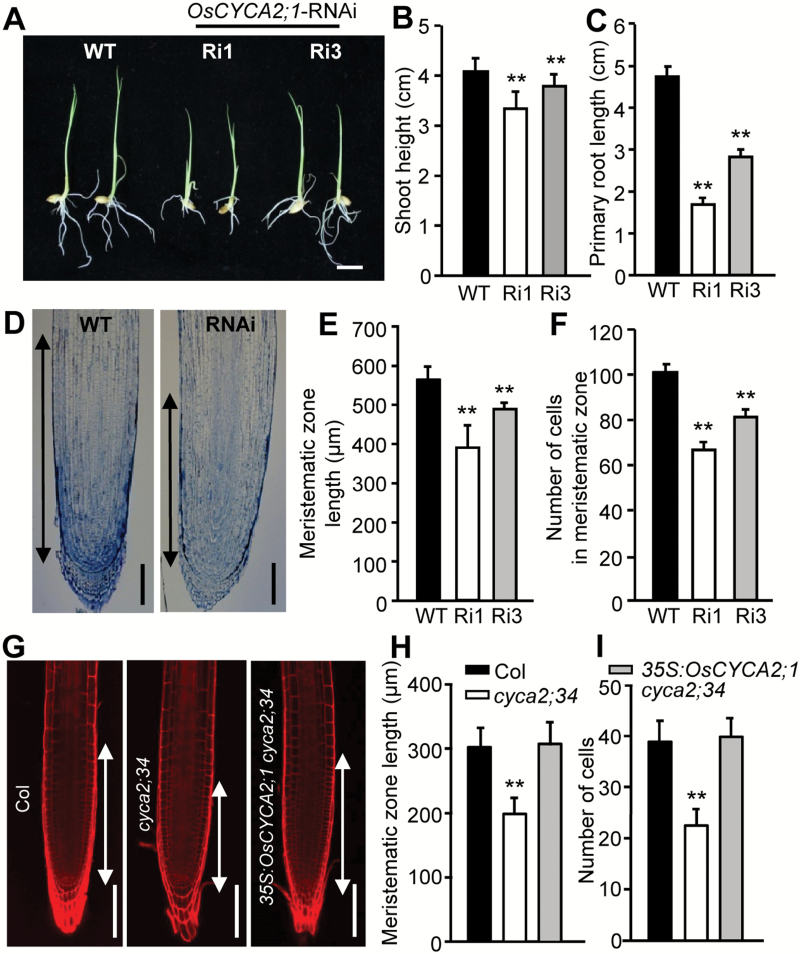
Suppression of *CYCA2* expression causes defective cell proliferation within the meristematic zone of roots. (A) Ten-day-old *OsCYCA2;1*-RNAi and wild-type (WT) rice seedlings. Scale bar=1 cm. (B, C) The length of shoots and primary roots (*n*=24). (D) Longitudinal sections of the primary root tips. Scale bar=100 µm. (E, F) Length and cell number of the meristematic zone in roots (*n*=20). (G) Propidium iodide-stained images of Arabidopsis root tips. Scale bar=100 µm. (H, I) Length and cell number of the meristematic zone in Arabidopsis roots (*n*=20). Double-headed arrows in (D, G) indicate the extent of the meristematic zone. Data in (B, C, E, F, H, I) represent the mean ±SD. Asterisks indicate a significant difference from WT controls (Student’s *t*-test, **P<0.01, *P<0.05). (This figure is available in colour at *JXB* online.)

*OsCYCA2;1* is preferentially expressed in proliferating tissues. Besides in the base dividing zone (proximal end) of leaves, a higher transcript level of *OsCYCA2;1* is found in rice root tips, implying that a similar *OsCYCA2;1* regulatory mechanism exists in rice roots (Supplementary Fig. 3B). Therefore, we probed the impact of down-regulated expression of *OsCYCA2;1* on root growth. As shown in Fig. 4, the overall growth of shoots and roots in 10-day-old *OsCYCA2;1*-RNAi transgenic seedlings is much less than in the wild type (Fig. 4A–C). To determine whether the root growth defects arose from a defective cell proliferation, we compared the root longitudinal sections of the wild type and the RNAi line. The shorter meristematic zone in *OsCYCA2;1*-RNAi is correlated with a considerably fewer number of cells within its meristematic zone (Fig. 4D–F). Similarly, Arabidopsis *cyca2;34* mutants exhibit a short meristematic zone and fewer cells than Col wild type. Ectopic expression of *OsCYCA2;1* restored the length of and cell number within the meristematic zone to the wild-type level (Fig. 4G–I), supporting that *OsCYCA2;1* is an evolutionarily conserved regulator that is required for cell proliferation in roots.

**Fig. 5.**
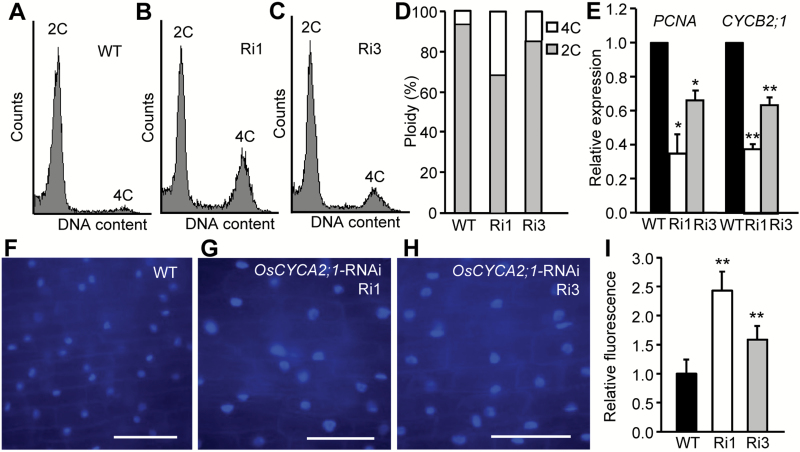
*OsCYCA2;1* is required for rice root cell mitosis. (A–C) Profiles of distribution of cells with different DNA content after flow cytometric analysis. Roots of *OsCYCA2;1*-RNAi lines Ri1 (B) and Ri3 (C) have more 4C cells than wild-type (WT) roots (A). (D) Quantitative analysis of the cell DNA ploidy levels. (E) Relative expression levels of *PCNA* and *CYCB2;1* in *OsCYCA2;1*-RNAi lines and WT roots. (F–H) DAPI staining of the epidermal cells in the maturation zone of the WT (F), *OsCYCA2;1*-RNAi line Ri1 (G), and Ri3 (H). Scale bar=50 µm. (I) Quantitative analysis of DAPI fluorescence revealed that *OsCYCA2;1*-RNAi transgenic lines have a higher average DNA content than the WT. Data represent the mean ±SD. Asterisks indicate a significant difference from WT controls (Student’s *t*-test, **P<0.01). (This figure is available in colour at *JXB* online.)

By means of flow cytometry approaches, we found that, in contrast to the 6% of 4C cells in the wild type, the fractions of cells with 4C DNA content in *OsCYCA2;1*-RNAi lines Ri1 and Ri3 are dramatically increased to 32% and 15%, respectively (Fig. 5A–D). Moreover, the relative expression levels of an S-phase-specific gene, *PCNA*, and a M-phase cyclin gene, *CYCB2;1*, were suppressed in *OsCYCA2;1*-RNAi plants (Fig. 5E). Consistently, epidermal cells in the maturation zone of *OsCYCA2;1*-RNAi roots showed stronger DAPI fluorescent signals than in the wild type (Fig. 5F–H). Quantitative analysis of the DAPI fluorescence intensities further confirmed that a higher DNA level (~2-fold) is present in *OsCYCA2;1*-RNAi roots (Fig. 5I). The higher DNA content in *OsCYCA2;1*-RNAi root cells might be due to delayed or arrested G_2_ to M transition, a result supporting the idea that *OsCYCA2;1* is required for cell mitosis.

### OsCYCA2;1 conservatively interacts with OsCDKB1;1

**Fig. 6.**
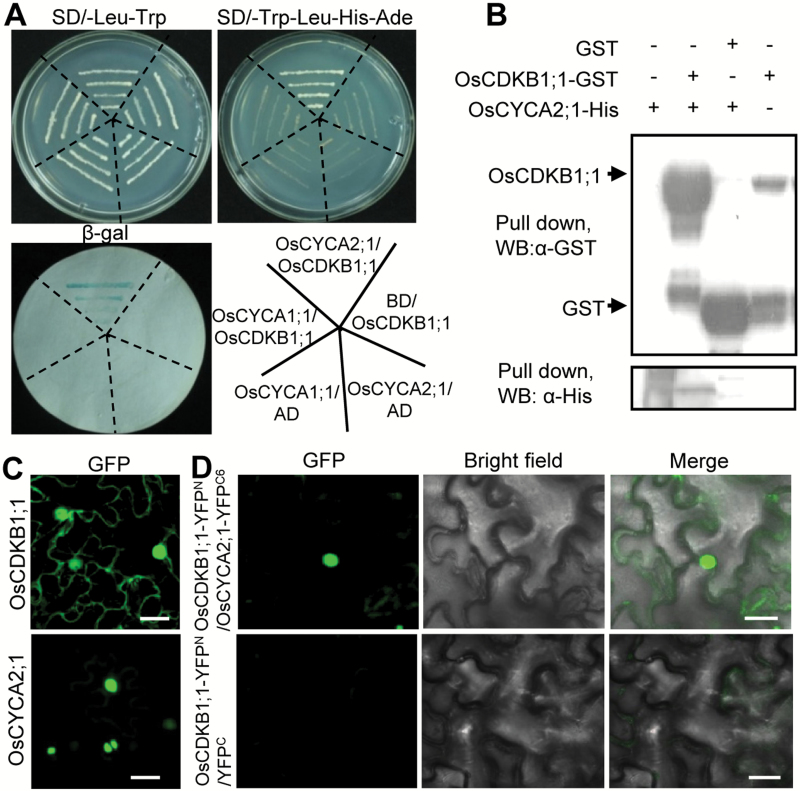
OsCYCA2;1 directly interacts with OsCDKB1;1. (A) Yeast two-hybrid assay. (B) Protein pull-down assay. (C) Transient expression of OsCDKB1;1–GFP and OsCYCA2;1–GFP in tobacco leaves. Scale bar=20 µm. (D) Bimolecular fluorescence complementation assay shows that OsCDKB1;1 interacts with OsCYCA2;1 in nuclei. Scale bar=20 µm. (This figure is available in colour at *JXB* online.)

CYCA2s play their regulatory roles through interacting with multiple CDKs, such as by forming CYCA2;3–CDKB1;1 or CYCA2;3–CDKA;1 protein complexes. Arabidopsis AtCYCA2;3 interacts with AtCDKB1;1 to form a functional complex which promotes the formation of a two-celled stoma and prevents entry into the endocycle program (Boudolf *et al.*, 2009; Vanneste *et al.*, 2011). According to the sequence blasting results in the rice genome, *Os01g67160* encodes the putative OsCDKB1;1. The deduced amino acid sequence of OsCDKB1;1 shares 88.5% sequence identity with the Arabidopsis CDKB1s. A B1-type-specific cyclin interaction motif ‘PPTALRE’ is highly conserved in rice OsCDKB1;1 (Supplementary Fig. S6). Yeast two-hybrid assays showed that OsCYCA2;1 can interact with OsCDKB1;1 (Fig. 6A). Consistently, pull-down assays verified the direct protein interaction between OsCYCA2;1 and OsCDKB1;1 (Fig. 6B). To determine the subcellular localization, OsCYCA2;1 or OsCDKB1;1 fused with GFP were transiently expressed in tobacco (*N. benthamiana*) leaves. The fluorescent signals from OsCYCA2;1–GFP were exclusively detected in nuclei, while OsCDKB1;1–GFP was found in both the cytoplasm and nuclei (Fig. 6C). BiFC analysis confirmed that OsCYCA2;1 directly interacts with OsCDKB1;1 in nuclei (Fig. 6D). These results suggest that OsCYCA2;1 may act as a conserved activator regulating the activity of OsCDKB1;1 kinase in rice.

### Suppression of OsCDKB1;1 has no obvious effects on rice development

To determine whether OsCDKB1;1, like its partner OsCYCA2;1, is required for rice development, we generated and selected two *OsCDKB1;1*-RNAi transgenic lines, Ri2 and Ri3, in which *OsCDKB1;1* transcript levels were significantly suppressed (Supplementary Fig. S7A). However, the overall growth of these two transgenic lines is comparable with that of the untransformed controls (Supplementary Fig. S7B–D). Longitudinal sections of roots demonstrate that the suppression of *OsCDKB1;1* has no significant impact on cell numbers of the root meristematic zone (Supplementary Fig. S7E–G). In addition, we found that neither the stomatal production (stomatal density) nor the stomatal complex morphology has been affected in *OsCDKB1;1*-RNAi transgenic lines (Supplementary Fig. S7H–J).

Flow cytometric assays also indicate that DNA ploidy levels were not changed in either the roots or shoots of *OsCDKB1;1*-RNAi (Supplementary Fig. S8A–H). Consistent with this, the expression of S-phase *PCNA* and M-phase cyclin *CYCB2;1* was not different between wild-type and *OsCDKB1;1*-RNAi transgenic plants (Supplementary Fig. S8I). Taken together, it seems that cell division was not interrupted by down-regulation of the *OsCDKB1;1* transcript level in transgenic rice, though we could not exclude the possibility that the remaining activity of OsCDKB1;1 protein is sufficient to function.

### *OsCYCA2;1* and *OsCDKB1;1* enable complementation of Arabidopsis cdkb1;1 1;2

**Fig. 7.**
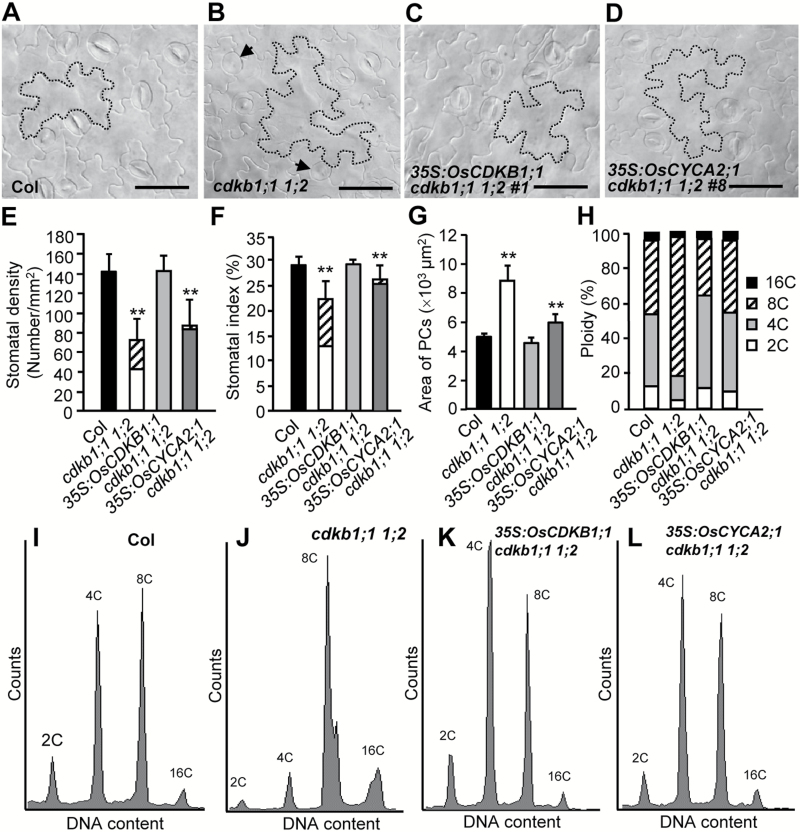
Ectopic expression of *OsCYCA2;1* and *OsCDKB1;1* complements Arabidopsis *cdkb1;1 1;2* mutant phenotypes. (A–D) DIC images of the epidermis of 14-day-old Arabidopsis cotyledons. Arrows indicate the formation of SGCs. Representative PCs are traced with dashed lines. Scale bar=50 µm. (E–G) Comparison of stomatal density, stomatal index, and area of PCs from the cotyledons. The diagonal line-filled box indicates the fraction of SGCs. Data represent the mean ±SD (*n*=24). Asterisks indicate a significant difference from Col after Student’s *t*-test, **P<0.01. (H) Proportions of cells with different ploidies. (I–L) Results from the flow cytometric analysis; ~10000 cell nuclei were measured for each sample.

Arabidopsis *cdkb1;1 1;2* mutants, like the *cyca2;34* mutants, display a decreased stomatal production, formation of SGCs, enlarged PCs, and increased cell ploidy levels (Boudolf *et al.*, 2004*a*; Xie *et al.*, 2010). Introduction of *OsCDKB1;1* fully complements the impaired GMC division in *cdkb1;1 1;2*, and restores stomatal production, indicating that *OsCDKB1;1* has the ability to promote both symmetric and asymmetric division. Meanwhile, expression of *OsCKDB1;1* could efficiently prevent the occurrence of enlarged PCs and increased DNA ploidy levels in *cdkb1;1 1;2* mutants (Fig. 7; Supplementary Figs S9, S10).

Interestingly, the defective stomatal production, impaired GMC division, and abnormal cell enlargement and DNA levels in *cdkb1;1 1;2* could be partially rescued by overexpression of *OsCYCA2;1* (Fig. 7; Supplementary Figs S9, S10). It is therefore possible that *OsCDKB1;1* and *OsCYCA2;1* have evolved from the common ancestor genes with Arabidopsis *AtCDKB1* genes and *AtCYCA2* genes, even though the developmental pathways of the two species have been diverged.

## Discussion

The control of cell division and differentiation is the core of the development and morphogenesis of multicellular organisms. Cyclins, known as conserved activators for the activity of CDKs, play a crucial regulatory role in cell cycle progression in diverse species. The functional pathway of CYCA2s and CDKB1s has been well investigated in the model plant Arabidopsis (Boudolf *et al.*, 2004*b*, 2009; Imai *et al.*, 2006; Xie *et al.*, 2010; Vanneste *et al.*, 2011; Yang *et al.*, 2014). In this study, we generated RNAi transgenic rice lines and performed cross-species complement tests to explore the function of the single rice A2-type cyclin, OsCYCA2;1, as well the single rice B1-type CDK, OsCDKB1;1. Cross-species expression of *OsCYCA2;1* or *OsCDKB1;1* enables rescue of the defective asymmetric entry divisions for stomatal initiation and GMC symmetric divisions for GC production in Arabidopsis *cyca2;34* and/or *cdkb1;1 1;2* mutants, suggesting that both *OsCYCA2;1* and *OsCDKB1;1* might have evolved from the common ancestor genes with Arabidopsis.

In Arabidopsis, asymmetric divisions generated the early stomatal precursor cells, meristemoids. Meristemoids then differentiate into GMCs after a cell fate change. In grasses, GMCs are created directly by stomata initiating asymmetric divisions (entry division) without the prior precursor stage of meristemoid. Orthologs of Arabidopsis stomatal transcriptional regulators SPCH, MUTE, FAMA, ICE1, and SCRM2 have been identified in grasses (Liu *et al.*, 2009; Vatén and Bergmann, 2012; Ran *et al.*, 2013; Chen *et al.*, 2017). Instead of a single copy in Arabidopsis, the rice genome has duplicated *SPCH* genes, *OsSPCH1* and *OsSPCH2*. Similar to the weak allele of Arabidopsis *spch*, the rice mutant *osspch2* exhibits a reduced number of stomata (Liu *et al.*, 2009). In Arabidopsis, SPCH heterodimerizes with SCRM/ICE1 or AtSCRM2 to promote stomatal lineage initiation (Kanaoka *et al.*, 2008; Horst *et al.*, 2015). In contrast, in the grass *B. distachyon*, *BdICE1* and *BdSCRM2* show a functional diversity in regulating stomatal pattern and morphology (Raissig *et al.*, 2016). It is already known that Arabidopsis SPCH activity or stability is modulated by multiple kinases, including MPKs, GSK3/BIN2, and CDKs (Lampard *et al.*, 2009; Gudesblat *et al.*, 2012; Kim *et al.*, 2012; Le *et al.*, 2014). Phosphorylation of Ser186 of SPCH, which might be the target residue of CDKs, positively regulates stomata production (Yang *et al.*, 2015). Thus, it will be interesting to establish if there is a conserved regulatory mechanism between CDK–cyclin and SPCH-ICE1/SCRM2 in grasses.

Besides the involvement in stomatal initiation, Arabidopsis AtCYCA2s and CDKB1s are synergistically required for the GMC symmetric division that is a prerequisite for the final stomatal development (Vanneste *et al.*, 2011). Arabidopsis and rice share common GMC–GC processing; GMCs divide symmetrically to produce the paired GCs of stomata, though the GC shapes are distinct. Thus, a role for OsCDKB1–OsCYCA2 in rice GMC divisions has been highly expected. However, suppression of OsCYCA2;1 transcription in rice by RNAi does not affect the rice GMC symmetric division. It has been identified that transcription of *CDKB1;1*, *CYCA2;3*, and *CDKA;1* in Arabidopsis is repressed by FOUR LIPS(FLP)/MYB88 MYB transcription factors during the GMC–GC transition stages (Xie *et al.*, 2010; Vanneste *et al.*, 2011; Yang *et al.*, 2014). FAMA, like FLP/MYB, also binds to the *CDKB1;1* promoter (Hachez *et al.*, 2011) to limit the GMC divisions to one. In contrast to the tumor-like phenotype in Arabidopsis *fama-1* mutants, the loss-of-function rice allele os*fama-1* did not undergo excessive division except the appearance of misshaped GCs and showing a lack of stomatal pores (Liu *et al.*, 2009). These observations suggest that GMC–GC differentiation is uncoupled from GMC division, in which the putative downstream FAMA/FLP/MYB88, CDKB1;1, and CYCA2;1 are not essential.

According to the phylogenetic analysis, CYCA2 and CDKB1 widely exist in diverse plant species, both in plants bearing stomata and in plants lacking stomata, indicating that CYCA2 and CDKB1 might function as fundamental regulators of the mitotic cell cycle, as well as outside stomatal development. High expression of *OsCYCA2;1* is associated with a high activity of cell proliferation, such as in the proximal end of leaves or root tips (Supplementary Fig. S3B).

Endoreduplication often occurs in cell types that undergo specialized differentiation. In Arabidopsis, the highly differentiated epidermal cells, such as mature PCs and trichomes, usually undergo multiple rounds of DNA replication without mitosis, resulting in polyploid cells (Burssens *et al.*, 2000). In contrast to the differentiated cells in Arabidopsis, polyploid cells in rice can only be found in the endosperm (Sabelli and Larkins, 2009). In *Drosophila*, it has been reported that cyclin A is one of the key components of chromosomal DNA replication that prevents re-initiation of DNA replication. Overexpression of *Drosophila* cyclin A caused a reduction in ploidy levels and inhibition of the endocycle (Hayashi and Yamaguchi, 1999). Here we found that the fraction of 4C cells remarkably increased in *OsCYCA2;1*-RNAi transgenic plants, while most cells keep a 2C DNA content. However, the expression levels of an S-phase gene *PCNA* and a M-phase gene *CYCB2;1* were suppressed in *OsCYCA2;1*-RNAi plants. Therefore, we speculated that the increase of 4C cells might be caused by the arrested G_2_ to M transition, similar to the observation in the rice knockdown lines of *OsCDKB2;1* (Endo *et al.*, 2012).

However, the *OsCDKB1;1*-RNAi transgenic rice plants, in which *OsCDKB1;1* transcript levels were significantly decreased, display phenotypes comparable with wild-type rice seedlings regarding the stomatal density, root cell division, and DNA content. CDKBs are plant-specific cyclin-dependent kinases that can be subdivided into two groups according to the different cyclin-binding motifs, namely ‘PPTALRE’ in CDKB1s and ‘PPTTLRE’ in CDKB2s (Joubès *et al.*, 2001). In Arabidopsis, each CDKB1 and CDKB2 subgroup contains two members (Vandepoele *et al.*, 2002). It has been predicted that the rice genome has a single *CDKB1* gene and a single *CDKB2* gene, encoding OsCDKB1;1 and OsCDKB2;1, respectively (Supplementary Fig. S11). However, the amino acid sequence alignment revealed that rice OsCDKB1;1 and OsCDKB2;1 share the same ‘PPTALRE’ cyclin-binding motif (Supplementary Fig. S12). Expression of *OsCDKB2;1* has been detected in the dividing region of the rice root apex (Umeda *et al.*, 1999*a*). Transcription of rice *OsCDKB2;1* is abundant during the G_2_ to M phase. Knockdown of the *OsCDKB2;1* gene in rice induces an increase of the 4C cell population (Umeda *et al.*, 1999*b*; Endo *et al.*, 2012). In addition, OsCDKB2;1 promotes cell division in the root meristem probably through the association with OsCYCB2s (Lee *et al.*, 2003). Thus, we cannot rule out that OsCDKB1;1 might function redundantly with OsCDKB2;1, such as forming active CDK–cyclin complexes via binding to the same type cyclins (i.e. OsCYCA2;1). Previous *in situ* hybridization results showed that both *OsCDKA;1* and *OsCDKA;2* are expressed in dividing root cells of rice (Umeda *et al.*, 1999*b*). Thus, further characterization of rice CDK–cyclin pairing and activity can help to reveal the regulatory mechanisms of cell division and differentiation during rice development.

## Supplementary data

Supplementary data are available at *JXB* online.

Table S1. List of primers used in this study.

Fig. S1. Amino acid sequence comparison of A2-type cyclins from rice and Arabidopsis.

Fig. S2. In contrast to dicot Arabidopsis, only one or two copies of genes encoding CYCA2 are found in monocot grasses.

Fig. S3. Relative expression of *OsCYCA2;1* in rice RNAi transgenic plants and in different tissues of wild-type plants.

Fig. S4. Overexpression of rice *OsCYCA2;1* suppresses the enhanced endoreduplication levels in Arabidopsis *cyca2;34*.

Fig. S5. Correlation between stomatal phenotypes and *OsCYCA2;1* expression levels in Arabidopsis *cyca2;34* mutants harboring *OsCYCA2;1*.

Fig. S6. Comparison of the amino acid sequence of OsCDKB1;1 with that of Arabidopsis CDKB1;1 and CDKB1;2.

Fig. S7. Suppression of *OsCDKB1;1* has no obvious impact on rice root and stomatal development.

Fig. S8. Suppression of *OsCDKB1;1* has no obvious impact on the distribution of DNA ploidy.

Fig. S9. Expression analysis of *OsCDKB1;1*-OE and *OsCYCA2;1*-OE transgenic plants in Arabidopsis *cdkb1* mutants.

Fig. S10. Ploidy distribution analysis of Arabidopsis *cdkb1;1 1;2* mutants carrying rice *OsCDKB1;1* or *OsCYCA2;1* genes.

Fig. S11. Phylogenetic tree of CDKB1 and CDKB2 in monocots.

Fig. S12. CDKB1 and CDKB2 contain the same cyclin-binding domain in most monocots.

Supplementary Table and Figures

## Abbreviations:

CYCA2: CYCLIN A2
CDKB1: CYCLIN-DEPENDENT KINASE B1
GC: guard cell
GMC: guard mother cell
PC: pavement cell
SGC: single guard cell.

## References

[CIT0001] Bergmann DC, Sack FD 2007 Stomatal development. Annual Review of Plant Biology 58, 163–181. 10.1146/annurev.arplant.58.032806.104023 17201685

[CIT0002] Boruc J, Van den Daele H, Hollunder J, Rombauts S, Mylle E, Hilson P, Inzé D, De Veylder L, Russinova E 2010 Functional modules in the Arabidopsis core cell cycle binary protein–protein interaction network. The Plant Cell 22, 1264–1280. 20407024 10.1105/tpc.109.073635 PMC2879739

[CIT0003] Boudolf V, Barrôco R, Engler Jde A, Verkest A, Beeckman T, Naudts M, Inzé D, De Veylder L 2004 *a* B1-type cyclin-dependent kinases are essential for the formation of stomatal complexes in *Arabidopsis thaliana*. The Plant Cell 16, 945–955. 15031414 10.1105/tpc.021774 PMC412868

[CIT0004] Boudolf V, Lammens T, Boruc J, et al 2009 CDKB1;1 forms a functional complex with CYCA2;3 to suppress endocycle onset. Plant Physiology 150, 1482–1493. 19458112 10.1104/pp.109.140269 PMC2705057

[CIT0005] Boudolf V, Vlieghe K, Beemster GT, Magyar Z, Torres Acosta JA, Maes S, Van Der Schueren E, Inzé D, De Veylder L 2004 *b* The plant-specific cyclin-dependent kinase CDKB1;1 and transcription factor E2Fa-DPa control the balance of mitotically dividing and endoreduplicating cells in Arabidopsis. The Plant Cell 16, 2683–2692. 15377755 10.1105/tpc.104.024398 PMC520964

[CIT0006] Burssens S, de Almeida Engler J, Beeckman T, Richard C, Shaul O, Ferreira P, Van Montagu M, Inzé D 2000 Developmental expression of the *Arabidopsis thaliana* CycA2;1 gene. Planta 211, 623–631. 11089674 10.1007/s004250000333

[CIT0007] Chater CC, Caine RS, Tomek M, et al 2016 Origin and function of stomata in the moss *Physcomitrella patens*. Nature Plants 2, 16179. 27892923 10.1038/nplants.2016.179 PMC5131878

[CIT0008] Chater CCC, Caine RS, Fleming AJ, Gray JE 2017 Origins and evolution of stomatal development. Plant Physiology 174, 624–638. 28356502 10.1104/pp.17.00183 PMC5462063

[CIT0009] Chen N, Xu Y, Wang X, Du C, Du J, Yuan M, Xu Z, Chong K 2011 OsRAN2, essential for mitosis, enhances cold tolerance in rice by promoting export of intranuclear tubulin and maintaining cell division under cold stress. Plant, Cell and Environment 34, 52–64. 10.1111/j.1365-3040.2010.02225.x 20825577

[CIT0010] Chen ZH, Chen G, Dai F, Wang Y, Hills A, Ruan YL, Zhang G, Franks PJ, Nevo E, Blatt MR 2017 Molecular evolution of grass stomata. Trends in Plant Science 22, 124–139. 27776931 10.1016/j.tplants.2016.09.005

[CIT0011] Cooper B, Hutchison D, Park S, Guimil S, Luginbühl P, Ellero C, Goff SA, Glazebrook J 2003 Identification of rice (*Oryza sativa*) proteins linked to the cyclin-mediated regulation of the cell cycle. Plant Molecular Biology 53, 273–279. 14750518 10.1023/b:plan.0000007001.30865.0f

[CIT0012] Dewitte W, Murray JA 2003 The plant cell cycle. Annual Review of Plant Biology 54, 235–264. 10.1146/annurev.arplant.54.031902.134836 14502991

[CIT0013] Dolezel J, Greilhuber J, Suda J 2007 Estimation of nuclear DNA content in plants using flow cytometry. Nature Protocols 2, 2233–2244. 17853881 10.1038/nprot.2007.310

[CIT0014] Donner TJ, Scarpella E 2013 Transcriptional control of early vein expression of CYCA2; 1 and CYCA2;4 in Arabidopsis leaves. Mechanisms of Development 130, 14–24. 22842098 10.1016/j.mod.2012.07.002

[CIT0015] Endo M, Nakayama S, Umeda-Hara C, Ohtsuki N, Saika H, Umeda M, Toki S 2012 CDKB2 is involved in mitosis and DNA damage response in rice. The Plant Journal 69, 967–977. 22092531 10.1111/j.1365-313X.2011.04847.x PMC3440594

[CIT0016] Fabian-Marwedel T, Umeda M, Sauter M 2002 The rice cyclin-dependent kinase-activating kinase R2 regulates S-phase progression. The Plant Cell 14, 197–210. 11826308 10.1105/tpc.010386 PMC150560

[CIT0017] Franks PJ, Farquhar GD 2007 The mechanical diversity of stomata and its significance in gas-exchange control. Plant Physiology 143, 78–87. 17114276 10.1104/pp.106.089367 PMC1761988

[CIT0018] Gudesblat GE, Schneider-Pizoń J, Betti C, et al 2012 SPEECHLESS integrates brassinosteroid and stomata signalling pathways. Nature Cell Biology 14, 548–554. 22466366 10.1038/ncb2471

[CIT0019] Hachez C, Ohashi-Ito K, Dong J, Bergmann DC 2011 Differentiation of Arabidopsis guard cells: analysis of the networks incorporating the basic helix–loop–helix transcription factor, FAMA. Plant Physiology 155, 1458–1472. 21245191 10.1104/pp.110.167718 PMC3046599

[CIT0020] Hayashi S, Yamaguchi M 1999 Kinase-independent activity of Cdc2/cyclin A prevents the S phase in the Drosophila cell cycle. Genes to Cells 4, 111–122. 10320477 10.1046/j.1365-2443.1999.00243.x

[CIT0021] Horst RJ, Fujita H, Lee JS, Rychel AL, Garrick JM, Kawaguchi M, Peterson KM, Torii KU 2015 Molecular framework of a regulatory circuit initiating two-dimensional spatial patterning of stomatal lineage. PLoS Genetics 11, e1005374. 26203655 10.1371/journal.pgen.1005374 PMC4512730

[CIT0022] Huang YW, Tsay WS, Chen CC, Lin CW, Huang HJ 2008 Increased expression of the rice C-type cyclin-dependent protein kinase gene, *Orysa;CDKC;1*, in response to salt stress. Plant Physiology and Biochemistry 46, 71–81. 18054244 10.1016/j.plaphy.2007.10.013

[CIT0023] Imai KK, Ohashi Y, Tsuge T, Yoshizumi T, Matsui M, Oka A, Aoyama T 2006 The A-type cyclin CYCA2;3 is a key regulator of ploidy levels in Arabidopsis endoreduplication. The Plant Cell 18, 382–396. 16415207 10.1105/tpc.105.037309 PMC1356546

[CIT0024] Joubès J, Lemaire-Chamley M, Delmas F, Walter J, Hernould M, Mouras A, Raymond P, Chevalier C 2001 A new C-type cyclin-dependent kinase from tomato expressed in dividing tissues does not interact with mitotic and G1 cyclins. Plant Physiology 126, 1403–1415. 11500540 10.1104/pp.126.4.1403 PMC117141

[CIT0025] Kanaoka MM, Pillitteri LJ, Fujii H, Yoshida Y, Bogenschutz NL, Takabayashi J, Zhu JK, Torii KU 2008 *SCREAM/ICE1* and *SCREAM2* specify three cell-state transitional steps leading to *Arabidopsis* stomatal differentiation. The Plant Cell 20, 1775–1785. 18641265 10.1105/tpc.108.060848 PMC2518248

[CIT0026] Kim TW, Michniewicz M, Bergmann DC, Wang ZY 2012 Brassinosteroid regulates stomatal development by GSK3-mediated inhibition of a MAPK pathway. Nature 482, 419–422. 22307275 10.1038/nature10794 PMC3292258

[CIT0027] La H, Li J, Ji Z, Cheng Y, Li X, Jiang S, Venkatesh PN, Ramachandran S 2006 Genome-wide analysis of cyclin family in rice (*Oryza sativa* L.). Molecular Genetics and Genomics 275, 374–386. 16435118 10.1007/s00438-005-0093-5

[CIT0028] Lampard GR, Lukowitz W, Ellis BE, Bergmann DC 2009 Novel and expanded roles for MAPK signaling in Arabidopsis stomatal cell fate revealed by cell type-specific manipulations. The Plant Cell 21, 3506–3517. 19897669 10.1105/tpc.109.070110 PMC2798322

[CIT0029] Le J, Zou J, Yang K, Wang M 2014 Signaling to stomatal initiation and cell division. Frontiers in Plant Science 5, 297. 25002867 10.3389/fpls.2014.00297 PMC4066587

[CIT0030] Lee J, Das A, Yamaguchi M, Hashimoto J, Tsutsumi N, Uchimiya H, Umeda M 2003 Cell cycle function of a rice B2-type cyclin interacting with a B-type cyclin-dependent kinase. The Plant Journal 34, 417–425. 12753582 10.1046/j.1365-313x.2003.01736.x

[CIT0031] Liu T, Ohashi-Ito K, Bergmann DC 2009 Orthologs of *Arabidopsis thaliana* stomatal bHLH genes and regulation of stomatal development in grasses. Development 136, 2265–2276. 19502487 10.1242/dev.032938

[CIT0032] Obaya AJ, Sedivy JM 2002 Regulation of cyclin–Cdk activity in mammalian cells. Cellular and Molecular Life Sciences 59, 126–142. 11846025 10.1007/s00018-002-8410-1 PMC11337483

[CIT0033] Olsen JL, Rouzé P, Verhelst B, et al 2016 The genome of the seagrass *Zostera marina* reveals angiosperm adaptation to the sea. Nature 530, 331–335. 26814964 10.1038/nature16548

[CIT0034] Qu X, Peterson KM, Torii KU 2017 Stomatal development in time: the past and the future. Current Opinion in Genetics & Biology 45, 1–9. 10.1016/j.gde.2017.02.001 28219014

[CIT0035] Raissig MT, Abrash E, Bettadapur A, Vogel JP, Bergmann DC 2016 Grasses use an alternatively wired bHLH transcription factor network to establish stomatal identity. Proceedings of the National Academy of Sciences, USA 113, 8326–8331. 10.1073/pnas.1606728113 PMC4961163 27382177

[CIT0036] Ran JH, Shen TT, Liu WJ, Wang XQ 2013 Evolution of the bHLH genes involved in stomatal development: implications for the expansion of developmental complexity of stomata in land plants. PLoS One 8, e78997. 24244399 10.1371/journal.pone.0078997 PMC3823973

[CIT0037] Raven JA 2002 Selection pressures on stomatal evolution. New Phytologist 153, 371–386. 10.1046/j.0028-646X.2001.00334.x 33863217

[CIT0038] Sabelli PA, Larkins BA 2009 The development of endosperm in grasses. Plant Physiology 149, 14–26. 19126691 10.1104/pp.108.129437 PMC2613697

[CIT0039] Serna L 2011 Stomatal development in Arabidopsis and grasses: differences and commonalities. International Journal of Developmental Biology 55, 5–10. 21425077 10.1387/ijdb.103094ls

[CIT0040] Swenson KI, Farrell KM, Ruderman JV 1986 The clam embryo protein cyclin A induces entry into M phase and the resumption of meiosis in *Xenopus* oocytes. Cell 47, 861–870. 2946420 10.1016/0092-8674(86)90801-9

[CIT0041] Umeda M, Iwamoto N, Umeda-Hara C, Yamaguchi M, Hashimoto J, Uchimiya H 1999 *a* Molecular characterization of mitotic cyclins in rice plants. Molecular Genetics and Genomics 262, 230–238. 10.1007/s004380051079 10517318

[CIT0042] Umeda M, Umeda-Hara C, Yamaguchi M, Hashimoto J, Uchimiya H 1999 *b* Differential expression of genes for cyclin-dependent protein kinases in rice plants. Plant Physiology 119, 31–40. 9880343 10.1104/pp.119.1.31 PMC32234

[CIT0043] Vandepoele K, Raes J, De Veylder L, Rouzé P, Rombauts S, Inzé D 2002 Genome-wide analysis of core cell cycle genes in Arabidopsis. The Plant Cell 14, 903–916. 11971144 10.1105/tpc.010445 PMC150691

[CIT0044] Vanneste S, Coppens F, Lee E, et al 2011 Developmental regulation of CYCA2s contributes to tissue-specific proliferation in Arabidopsis. EMBO Journal 30, 3430–3441. 21772250 10.1038/emboj.2011.240 PMC3160660

[CIT0045] Vatén A, Bergmann DC 2012 Mechanisms of stomatal development: an evolutionary view. EvoDevo 3, 11. 22691547 10.1186/2041-9139-3-11 PMC3390899

[CIT0046] Walter M, Chaban C, Schütze K, et al 2004 Visualization of protein interactions in living plant cells using bimolecular fluorescence complementation. The Plant Journal 40, 428–438. 15469500 10.1111/j.1365-313X.2004.02219.x

[CIT0047] Wang G, Kong H, Sun Y, Zhang X, Zhang W, Altman N, DePamphilis CW, Ma H 2004 Genome-wide analysis of the cyclin family in Arabidopsis and comparative phylogenetic analysis of plant cyclin-like proteins. Plant Physiology 135, 1084–1099. 15208425 10.1104/pp.104.040436 PMC514142

[CIT0048] Xie Z, Lee E, Lucas JR, Morohashi K, Li D, Murray JA, Sack FD, Grotewold E 2010 Regulation of cell proliferation in the stomatal lineage by the Arabidopsis MYB FOUR LIPS via direct targeting of core cell cycle genes. The Plant Cell 22, 2306–2321. 20675570 10.1105/tpc.110.074609 PMC2929110

[CIT0049] Yang K, Wang H, Xue S, Qu X, Zou J, Le J 2014 Requirement for A-type cyclin-dependent kinase and cyclins for the terminal division in the stomatal lineage of Arabidopsis. Journal of Experimental Botany 65, 2449–2461. 24687979 10.1093/jxb/eru139 PMC4036514

[CIT0050] Yang KZ, Jiang M, Wang M, Xue S, Zhu LL, Wang HZ, Zou JJ, Lee EK, Sack F, Le J 2015 Phosphorylation of serine 186 of bHLH transcription factor SPEECHLESS promotes stomatal development in Arabidopsis. Molecular Plant 8, 783–795. 25680231 10.1016/j.molp.2014.12.014

[CIT0051] Yoshizumi T, Tsumoto Y, Takiguchi T, Nagata N, Yamamoto YY, Kawashima M, Ichikawa T, Nakazawa M, Yamamoto N, Matsui M 2006 Increased level of polyploidy1, a conserved repressor of CYCLINA2 transcription, controls endoreduplication in Arabidopsis. The Plant Cell 18, 2452–2468. 17012601 10.1105/tpc.106.043869 PMC1626625

