# Supplementary material for: A2-type cyclin is required for the asymmetric entry division in rice stomatal development
Source: J Exp Bot. 2018 Apr 26;69(15):3587–99. doi: 10.1093/jxb/ery158 (PMC6022656; doi:10.1093/jxb/ery158)
Supplement: Supplementary Table and Figures [file ery158_suppl_supplementary_tables_figures.pdf]

Table S1. Primers used in this study.

Purpose	Gene	Locus ID	Forward Primer (5'-3')	Reverse Primer (5'-3')
OE construct	*OsCYCA2;1*	LOC_Os12g31810	ATGGAGAAGTACGAGAAGCTG	TCAGCTGAAGAGTGACTGCC
	OsCDKB1;1	LOC_Os01g67160	ATGGAGAAGTACGAGAAGCTG	CTAGAACTGGGACTTGTCGAG
RNAi construct	*OsCYCA2;1*	LOC_Os12g31810	GCGTCGACATCGATGAGGCATTCTCATTGATTGG	GGGGTACCTCTAGACTTCAACACGAGGAGCACATA
	OsCDKB1;1	LOC_Os01g67160	GCGTCGACATCGATTGTGCTTCGTACTCAAATTGC	GGGGTACCTCTAGAGGAGTTCCCAACAACCTGAA
Pull down	*OsCYCA2;1*	LOC_Os12g31810	GGAATTCATGGCTGGAAGGAAGGAAAATC	CCTCGAGGCTGAAGAGTGACTGCC
	OsCDKB1;1	LOC_Os01g67160	GGAATTCTCTAGAATGGAGAAGTACGAGAAGCTG	CCTCGAGGAACTGGGACTTGTCGAG
	OsCYCA1;1	LOC_Os01g13260	CGGAATCCATGTCGAGCAACCTAGCAGC	CCGCTCGAGGCATGTTGCGTCGCGAA
BiFC/Subcellular location	*OsCYCA2;1*	LOC_Os12g31810	GCTCTAGAATGGCTGGAAGGAAGGAAAATC	GGGGTACCGCTGAAGAGTGACTGCC
	OsCDKB1;1	LOC_Os01g67160	GCTCTAGAATGGAGAAGTACGAGAAGCTG	GGGGTACCGAACTGGGACTTGTCGAG
	OsCYCA1;1	LOC_Os01g13260	GCTCTAGATGTCGAGCAACCTAGCAGC	GGGGTACCGCATGTTGCGTCGCGAA
RT-qPCR	*OsACTIN*	LOC_Os03g50885	GGATCCATCTTGGCATCTCTCA	GGGCCAGACTCGTCGTACTC
	OsCYCA2;1	LOC_Os12g31810	GAGGCATTCTCATTGATTGG	CTTCAACACGAGGAGCACAT
	OsCDKB1;1	LOC_Os01g67160	TTGCTGATCTTGGGCTAGG	GAAGATGCAACCAACGGAC

Underlined bases show restriction enzyme recognition sites.

Supplementary Figure S1

```
                                                                                        ****
OsCYCA2;1   MAGRKENPVLTACQAPSGRITRAQAAANRGRFGFAPSVSLPARTERKQTAKGKTKRGALDEITSASTATSAPQP.KRRTV   79
AtCYCA2;1                      MHRASSKHTNAKKEAISTSKIRDNNVRVTRSRAK.ALG....VSNSPSKPAFKHETKRVAR   56
AtCYCA2;2                      MYCSSSMHPNANKENISTSDVQESFVRITRSRAKKAMGRG..VSIPPTKPSFKQQ.KRRAV   58
AtCYCA2;3    MGKENAVS........RPFTRSLASALRAS.......EVTSTTQNQQRVN...TKR....PALEDTRATGPNKRKKRAV   57
AtCYCA2;4    MGKENAVSGNSIPIHGRPVTRALASALRASSKLITSSEVAATTQNQGRVLRAKSKR....TALDEKKANAP...KKRAV   72
Consensus     g                  tra a a ra    i s v   t r  r   k   g           tk  p   krrav

            *****
OsCYCA2;1   LKDVTNIGCA.....NSSKNCTTTSKLQQKSKPTQRVKQIPSKKQCAKKVPKLPPPAVAGTSFVIDSKSSEETQKVELLA  154
AtCYCA2;1   PSNKR..........MASDNITVCNQKRRAV........LKDVTNTLAESIISTEGNVK..ACKRGGKETKQIEEDGLVD  116
AtCYCA2;2   LKDVS..........NTSADIIYSELRKGGN........IKANRKCLKE............PKKAAKEGANSAMDILVD  107
AtCYCA2;3   LGEITNVNSN.....TAILEAKNSKQIKKGRG......HGLASTSQLATSVTSEVTDLQ...SRTDAKVEVASNTAGN..  121
AtCYCA2;4   LKDITNVTCENSYTSCFSVAVENIKQIKKGR.......QSSSSSKVASSSATSQVT.........DAKVEVVSNSAGASL  136
Consensus   lkd tn            s       q k g                l  s  s             dak    s  gl

OsCYCA2;1   KAEEPTNLFENEGLLSLQNIERNRDSNCHEAFFEARNAMDKHELADSKPGDS...SGLGFIDIDNDNGNPQMCASYASEI  231
AtCYCA2;1   VDGEKSKLAED...LSKIRMVESLDASAS.............KQKEDRSDVT...DCVQIVDIDSGVQDPQFCSLYAASI  177
AtCYCA2;2   MHTEKSKLAED...LSKIRMAEAQDVSLSNFKDEEI.....TEQQEDGSGVM...ELLQVVDIDSNVEDPQCCSLYAADI  176
AtCYCA2;3   ...LSVSKGTDNTADNCIEIWNSRLPPRPLGRSAS......TAEKSAVIGSSTVPDIPKFVDIDSDDKDPLLCCLYAPEI  192
AtCYCA2;4   SVFTDTSLGTNETSYSIIAKPSSRSPPRPFG..........TVERSCGGASS.....PKFVDIDSDDKDPLLCSLYAPDI  201
Consensus      e   l ed   ls i    srd                 t       g s      fvdidsd  dpq cslya  i

OsCYCA2;1   YTNLMASELIRRPRSNYMEALQRDITKGMRGILIDWLVEVSEEYKLVPDTLYLTINLIDRFLSQHYIERQKLQLLGITSM  311
AtCYCA2;1   YDSINVAELEQRPSTSYMVQVQRDIDPTMRGILIDWLVEVSEEYKLVSDTLYLTVNLIDRFMSHNYIEKQKLQLLGITCM  257
AtCYCA2;2   YDNIHVAELQQRPLANYMELVQRDIDPDMRKILIDWLVEVSDDYKLVPDTLYLTVNLIDRFLSNSYIERQRLQLLGVSCM  256
AtCYCA2;3   HYNLRVSELKRRPLPDFMERIQKDVTQSMRGILVDWLVEVSEEYTLASDTLYLTVYLIDWFLHGNYVQRQQLQLLGITCM  272
AtCYCA2;4   YYNLRVAELKRRPFPDFMEKTQRDVTETMRGILVDWLVEVSEEYTLVPDTLYLTVYLIDWFLHGNYVERQRLQLLGITCM  281
Consensus   y nl vael rrp   yme  qrdit  mrgilidwlvevseeyklvpdtlyltvnlidrfls nyierq lqllgitcm

OsCYCA2;1   LIASKYEEICAPRVEEFCFITDNTYTKAEVLKMEGLVLNDMGFHLSVPTTKTFLRRFLRAAQASRNVPSITLGYLANYLA  391
AtCYCA2;1   LIASKYEEISAPRLEEFCFITDNTYTRLEVLSMEIKVLNSLHFRLSVPTTKTFLRRFIRAAQASDKVPLIEMEYLANYFA  337
AtCYCA2;2   LIASKYEELSAPGVEEFCFITANTYTRPEVLSMEIQILNFVHFRLSVPTTKTFLRRFIKAAQASYKVPFIELEYLANYLA  336
AtCYCA2;3   LIASKYEEISAPRIEEFCFITDNTYTRDQVLEMENQVLKHFSFQIYTPTPKTFLRRFLRAAQASRLSPSLEVEFLASYLT  352
AtCYCA2;4   LIASKYEEIHAPRIEEFCFITDNTYTRDQVLEMESQVLKHFSFQIYTPTSKTFLRRFLRAAQVSFPNQSLEMEFLANYLT  361
Consensus   liaskyeeisapr eefcfitdntytr evl me qvln   f lsvpttktflrrflraaqas  vpsie eylanyla

OsCYCA2;1   ELTLIDYSFLKFLPSVVAASAVFLARWTLDQSDIPWNHTLEHYTSYKSSDIQICVCALRELQHNTSNCPLNAIREKYRQQ  471
AtCYCA2;1   ELTLTEYTFLRFLPSLIAASAVFLARWTLDQSNHPWNQTLQHYTRYETSALKNTVLAMEELQLNTSGSTLIAIHTKYNQQ  417
AtCYCA2;2   ELTLVEYSFLRFLPSLIAASAVFLARWTLDQTDHPWNPTLQHYTRYEVAELKNTVLAMEDLQLNTSGCTLAATREKYNQP  416
AtCYCA2;3   ELTLIDYHFLKFLPSVVAASAVFLAKWTMDQSNHPWNPTLEHYTTYKASDLKASVHALQDLQLNTKGCPLSAIRMKYRQE  432
AtCYCA2;4   ELTLMDYPFLKFLPSIIAASAVFLAKWTLNQSSHPWNPTLEHYTTYKASDLKASVHALQDLQLNTKGCSLNSIRMKYRQD  441
Consensus   eltl dy flkflps iaasavflarwtldqs hpwnptlehyt yk sdlk  v al dlqlntsgc l air kyrq

OsCYCA2;1   KFECVANLTSPELGQSLFS                                                              490
AtCYCA2;1   KFKRVATLTSPERVNTLFS                                                              436
AtCYCA2;2   KFKSVAKLTSPKRVTSLFS                                                              435
AtCYCA2;3   KYKSVAVLTSPKLLDTLF                                                               450
AtCYCA2;4   KFKSVAVFSSGELPDKLFI                                                              460
Consensus   kfksva ltspel   lfs
```

Fig. S1. Amino acid sequence comparison of A2-type cyclins from rice and *Arabidopsis.*

Comparison of the predicted amino acid sequences of rice OsCYCA2;1 with *Arabidopsis* AtCYCA2;1, AtCYCA2;2, AtCYCA2;3, and AtCYCA2;4. The dashed line boxes indicate cyclin box. Amino acid residues with black backdrop indicate identity, and those with grey backdrop indicate conserved substitutions.

Supplementary Figure S2

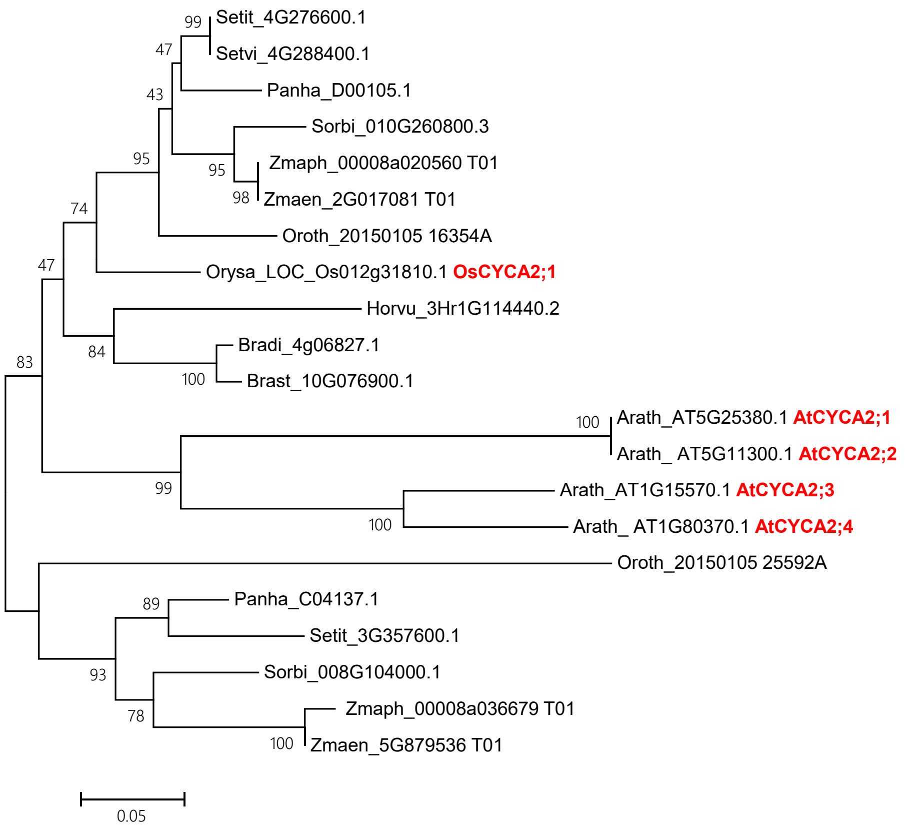

Fig. S2. In contrast to dicot *Arabidopsis*, only one or two copies of genes encoding CYCA2 are found in monocot grasses.

Phylogenetic tree using amino acid sequences of selected OsCYCA2;1 based on Phytozome V12.1, using the neighbor-joining method on MEGA4. Bootstrap values for 1000 replicates are given in nodes as percents. Amino acid sequences from *Arabidopsis thaliana*, *Brachypodium stacei*, *Brachypodium distachyon*, *Hordeum vulgare*, *Oropetium thomaeum*, *Oryza sativa*, *Panicum hallii*, *Sorghum bicolor*, *Setaria italica*, *Setaria viridis*, *Zea mays ph207*, and *Zea mays Ensembl* were used to generate the tree. Underlined letters used for the species abbreviations in the tree.

Supplementary Figure S3

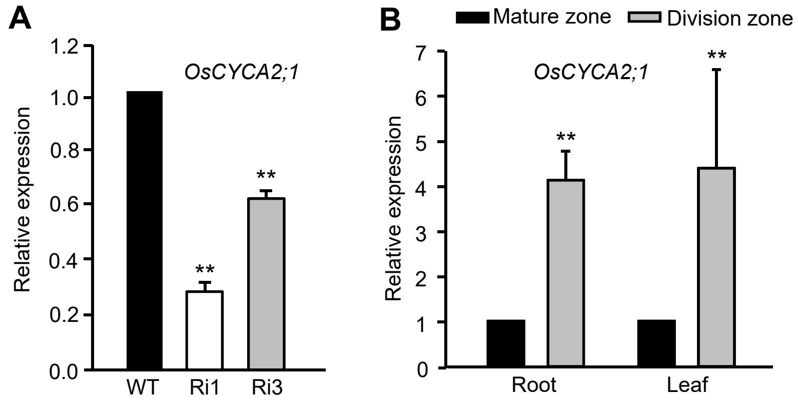

Fig. S3. Relative expression of *OsCYCA2;1* in RNA interference transgenic plants and in rice different tissues of wild-type plants.

(A) Relative OsCYCA2;1 transcript levels were suppressed in the rice *OsCYCA2;1-RNAi* lines Ri1 and Ri3. (B) Relative *OsCYCA2;1* transcript levels in different tissues of WT plants. The division zone and mature zone of roots and leaves were analyzed. Asterisks indicate significant difference to mature zone. Date are mean $\pm$ SD. (Student's *t*-test, $^{**}P< 0.01$).

Supplementary Figure S4

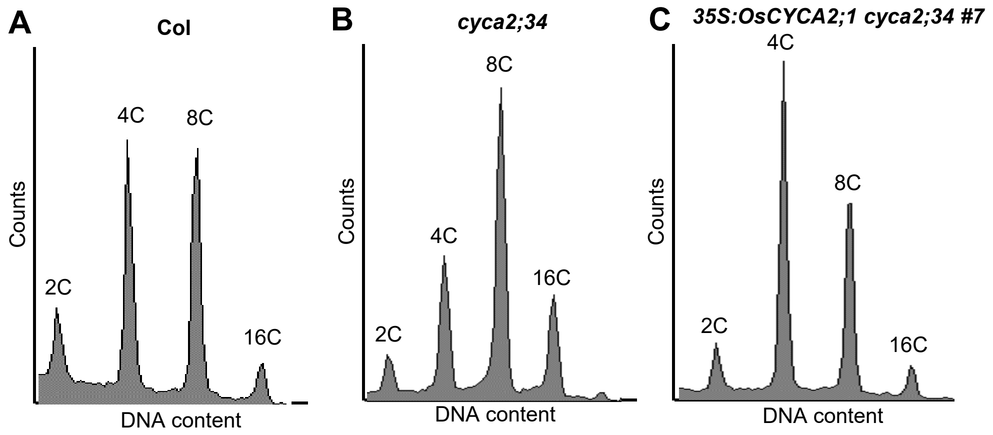

Fig. S4. Overexpression of rice *OsCYCA2;1* suppresses the enhanced endoreduplication levels in *Arabidopsis cyca2;34*.

Distribution of shoot cells with different DNA content, revealed by flow cytometric analysis. (A) Col. (B) *cyca2;34* shows an increase in the 8C and 16C peaks. (C) The profile in *35S:OsCYCA2;1 cyca2;34* (line #7) is similar to Col, indicating that the overexpression of *OsCYCA2;1* inhibits endoreduplication in *Arabidopsis* shoots. For each line ~10,000 cell nuclei were measured.

Supplementary Figure S5

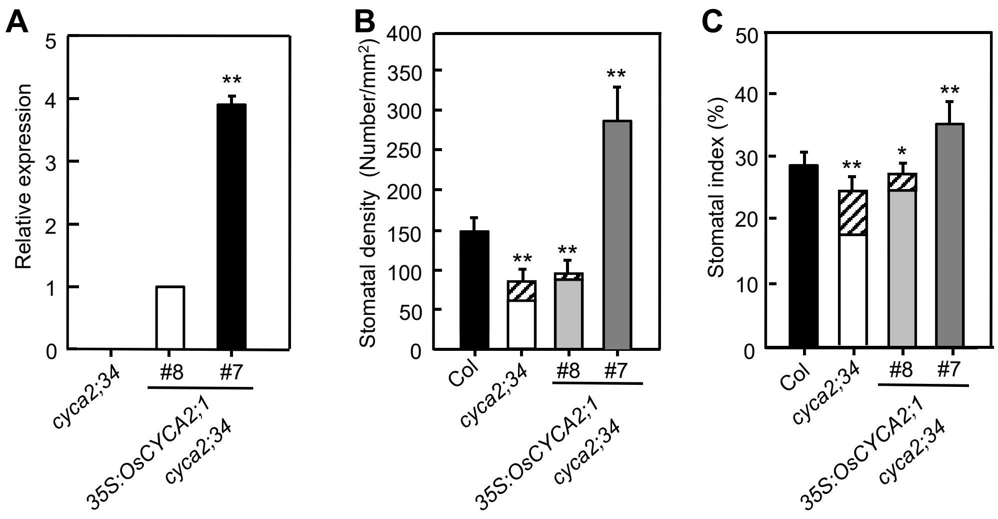

Fig. S5. Correlation between stomatal phenotypes and *OsCYCA2;1* overexpression levels in *Arabidopsis cyca2;34* mutants harboring *OsCYCA2;1*.

(A) RT-qPCR analysis of two representative *cyca2;34* harboring *35S:OsCYCA2;1* lines with low (line #8) and high (line #7) *OsCYCA2;1* transcript levels. Asterisks indicate significant difference between two lines (Student's *t*-test, ***P*< 0.01). (B) Stomatal density and index of cotyledon in Col, *cyca2;34*, and *cyca2;34* harboring *35S:OsCYCA2;1*. The diagonal line-filled box indicates SGC. Data represent mean ± SD. Asterisks indicate significant difference to the Col (Student's *t*-test, ***P*< 0.01, **P*< 0.05).

Supplementary Figure S6

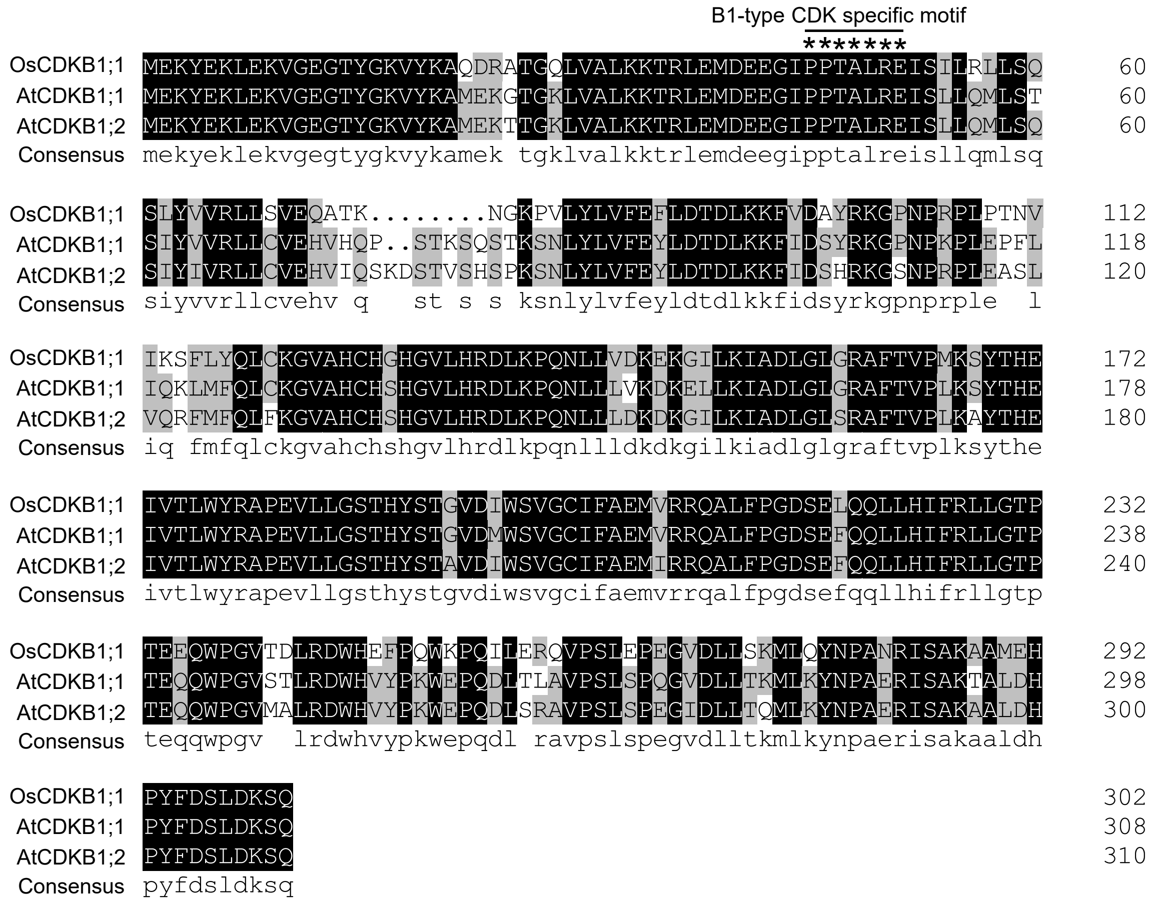

Fig. S6. Comparison of amino acid sequence of OsCDKB1;1 with *Arabidopsis* CDKB1;1 and CDKB1;2.

Alignment of the predicated rice OsCDKB1;1 protein with *Arabidopsis* AtCDKB1;1 and AtCDKB1;2. The amino acid sequences marked with asterisks is B1-type specific cyclin interaction motif ‘PPTALRE’.

Supplementary Figure S7

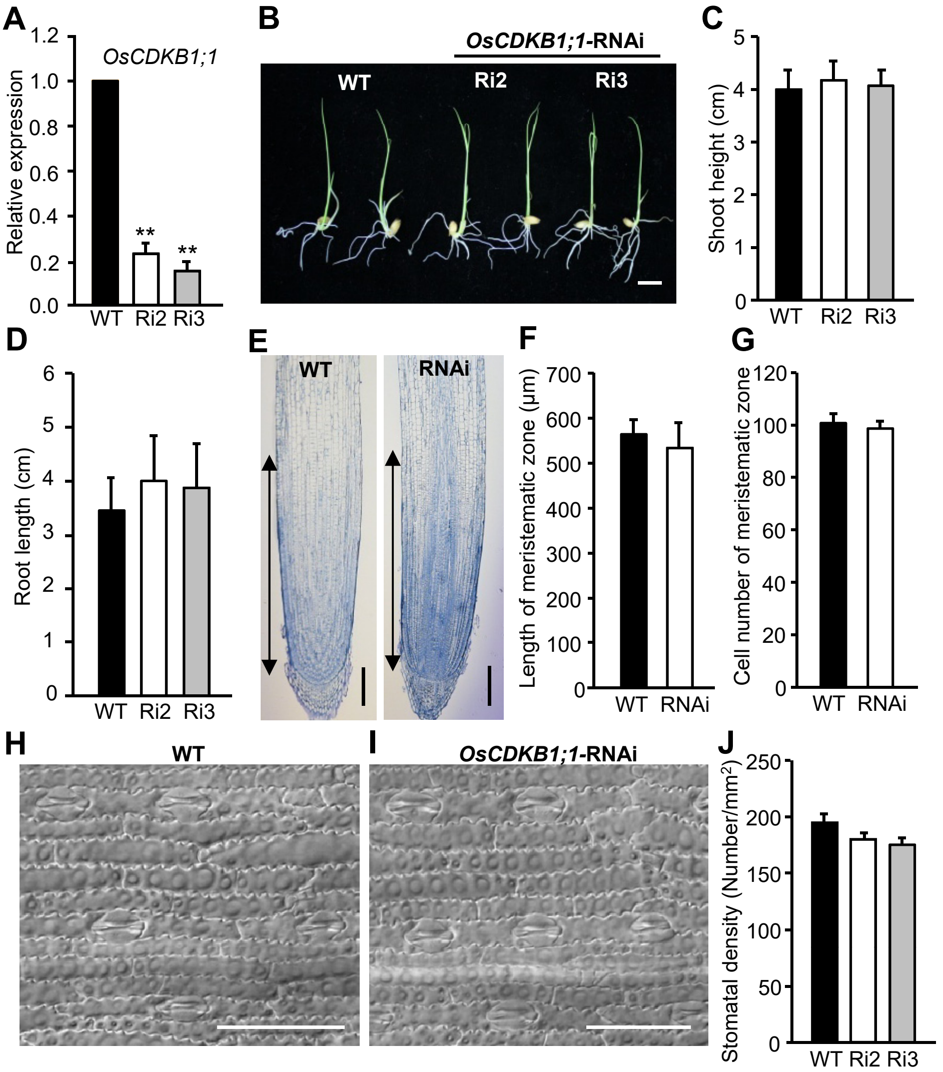

Fig. S7. Suppression of *OsCDKB1;1* has no obvious impact on rice root and stomatal development.

(A) *OsCDKB1;1* transcript level was significantly suppressed in *OsCDKB1;1*-RNAi transgenic plants, lines Ri2 and Ri3. (B) Ten-day-old *OsCDKB1;1*-RNAi and WT seedlings. Scale bar = 1 cm. (C) No significant difference in shoot length was found between *OsCDKB1;1*-RNAi and WT seedlings (*n* = 48). (D) No significant difference in root length was found between *OsCDKB1;1*-RNAi and WT seedlings (*n* = 48). (E) Longitudinal sections of primary root tips of 6-day-old *OsCDKB1;1*-RNAi and WT seedlings. Scale bar = 100 μm. (F and G) Length and cell number of the meristematic zone in roots of *OsCDKB1;1*-RNAi and WT plants. (*n* = 24). (H and I) DIC images of rice leaf epidermis from 10-day-old WT and *OsCDKB1;1*-RNAi plants. Scale bar = 50 μm. (J) No significant difference of stomatal density was found between *OsCDKB1;1*-RNAi and WT mature leaves (*n* = 30). Data in (A,C,D,F,J) represent mean ± SD. Statistic analysis performed after Student's *t*-test, ***P*< 0.01.

Supplementary Figure S8

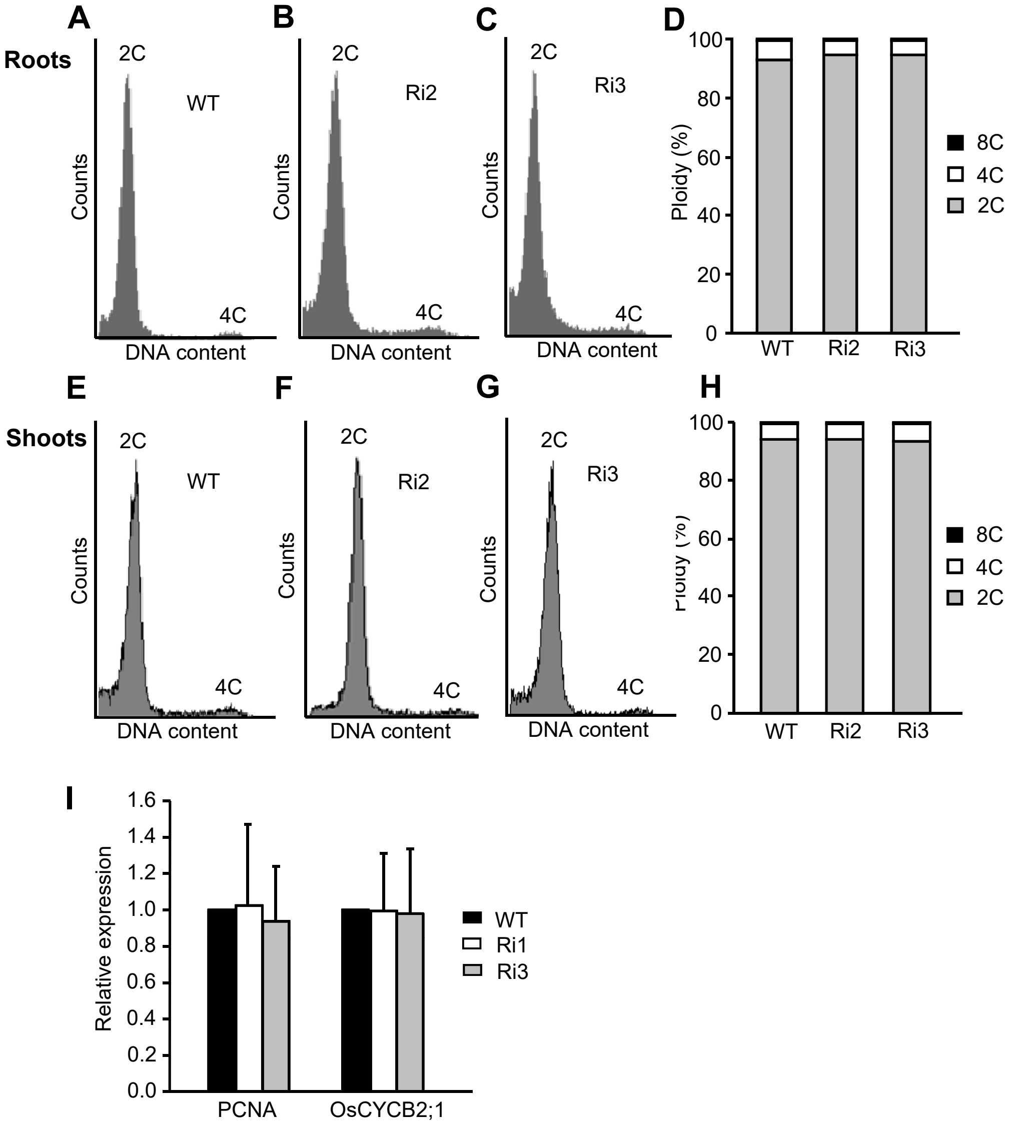

Figure S8. Suppression of *OsCDKB1;1* has no obvious impact on the distribution of DNA ploidy.

(A-C) Flow cytometric analysis of cell nuclei in roots cells of WT (A), *OsCDKB1;1*-RNAi lines Ri2 (B) and Ri3 (C). (D) Proportions of 4C and 8C cells in roots of two *OsCDKB1;1*-RNAi lines and WT. (E-G) Flow cytometric analysis of cell nuclei in shoots of WT (E), *OsCDKB1;1*-RNAi lines Ri1 (F) and Ri2 (G). (H) Proportions of 4C and 8C cells in the shoot of two *OsCDKB1;1*-RNAi lines and WT. For each line ~10,000 cell nuclei were measured. (I) The relative expression levels of *PCNA* and *OsCYCB2;1* are not changed in *OsCDKB1;1*-RNAi lines. Data represent mean ± SD. Statistic analysis performed after Student's *t*-test.

Supplementary Figure S9

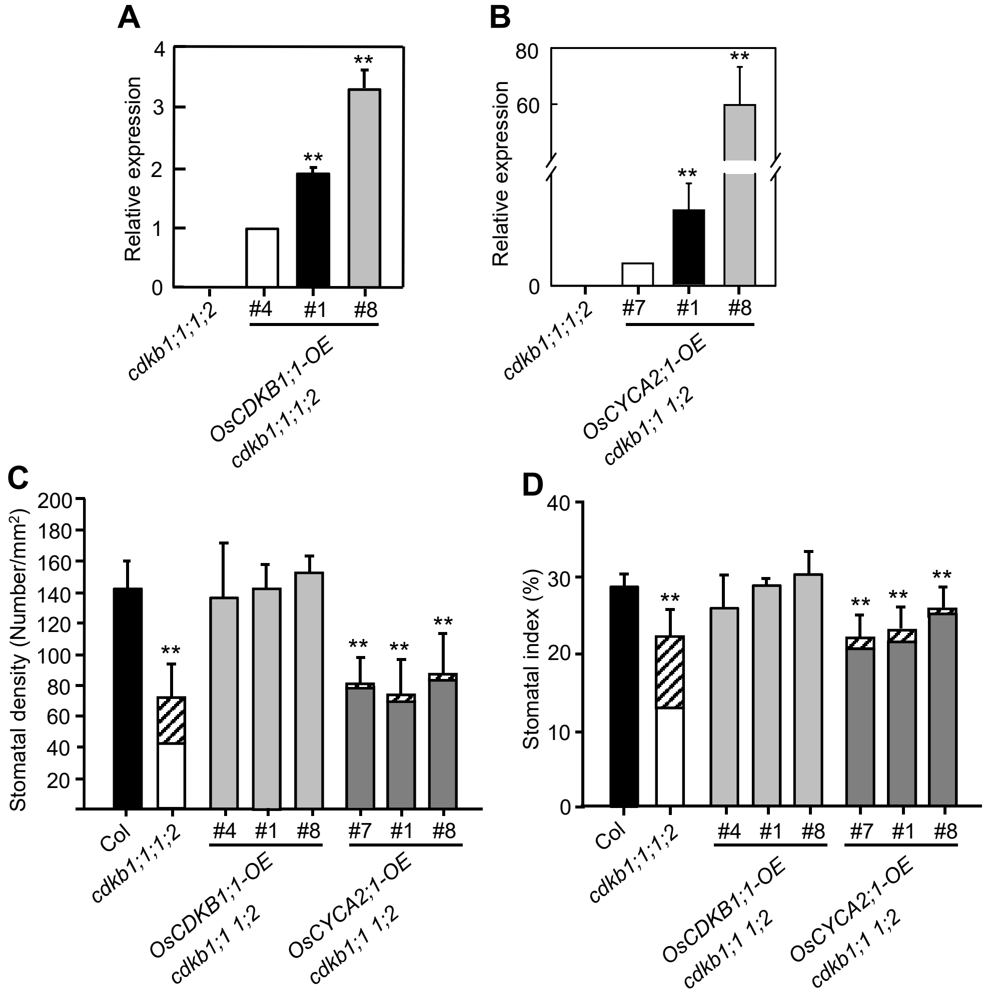

Fig. S9. Expression analysis of *OsCDKB1;1*-OE and *OsCYCA2;1*-OE transgenic plants in *Arabidopsis cdkb1* mutants.

(A) RT-qPCR analysis *OsCDKB1;1* transcripts levels in *OsCDKB1;1*-OE *cdkb1;11;2* plants. (B) The expression levels *OsCYCA2;1* in *OsCYCA2;1*-OE *cdkb1;11;2* plants. Asterisks indicate significant difference to *cdkb1;11;2* mutants. (C) Stomatal density and index of cotyledon in Col, *cdkb1;11;2*, *OsCDKB1;1*-OE *cdkb1;11;2* and *OsCYCA2;1*-OE *cdkb1;11;2* plants. The diagonal line-filled box indicates SGC. Data represent mean ± SD. Asterisks indicate significant difference to the Col (Student's *t*-test, ***P*< 0.01).

Supplementary Figure S10

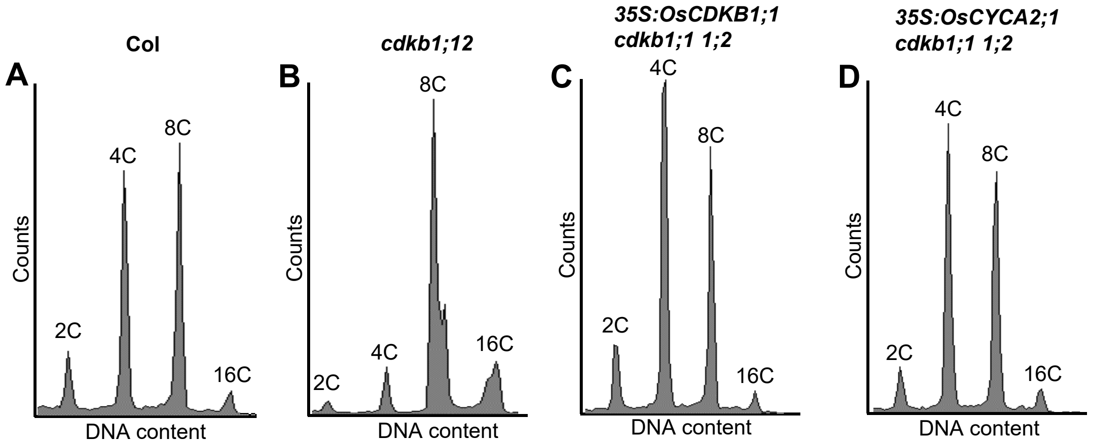

Figure S10. Ploidy distribution analysis of *Arabidopsis* cdkb1;1 1;2 mutants carrying rice *OsCDKB1;1* or *OsCYCA2;1* genes
(A-D) Flow cytometry analysis showing that shoot cells ploidy distribution of Col, *cdkb1;11;2, 35S:OsCDKB1;1 cdkb1;1 1;2*, and *35S:OsCYCA2;1 cdkb1;1 1;2* plants. For each line ~10,000 cell nuclei were measured.

Supplementary Figure S11

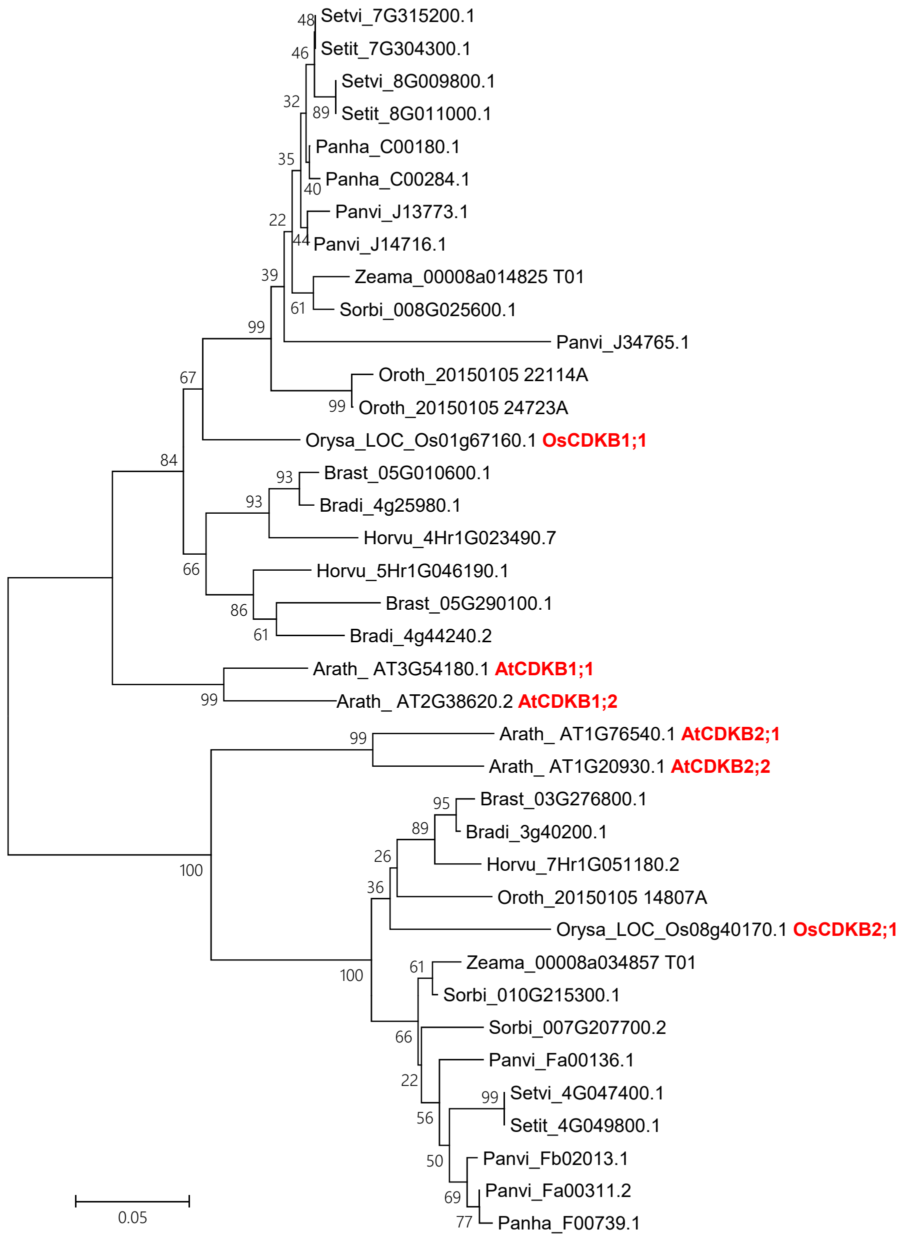

Fig. S11. Phylogenetic tree of CDKB1 and CDKB2 in monocots.

Phylogenetic trees constructed using amino acid sequences of selected OsCDKB1;1 gene family members based on Phytozome V12.1, using the neighbour-joining method on MEGA4. Bootstrap values for 1000 replicates are given in nodes as percents. Amino acid sequences from *Arabidopsis thaliana*, *Brachypodium stacei*, *Brachypodium distachyon*, *Hordeum vulgare*, *Oryza sativa*, *Oropetium thomaeum*, *Panicum hallii*, *Panicum virgatum*, *Sorghum bicolor*, *Setaria italica*, *Setaria viridis*, and *Zea mays*, were used to generate trees. Underlined letters used for the species abbreviations in the tree.

Supplementary Figure 12

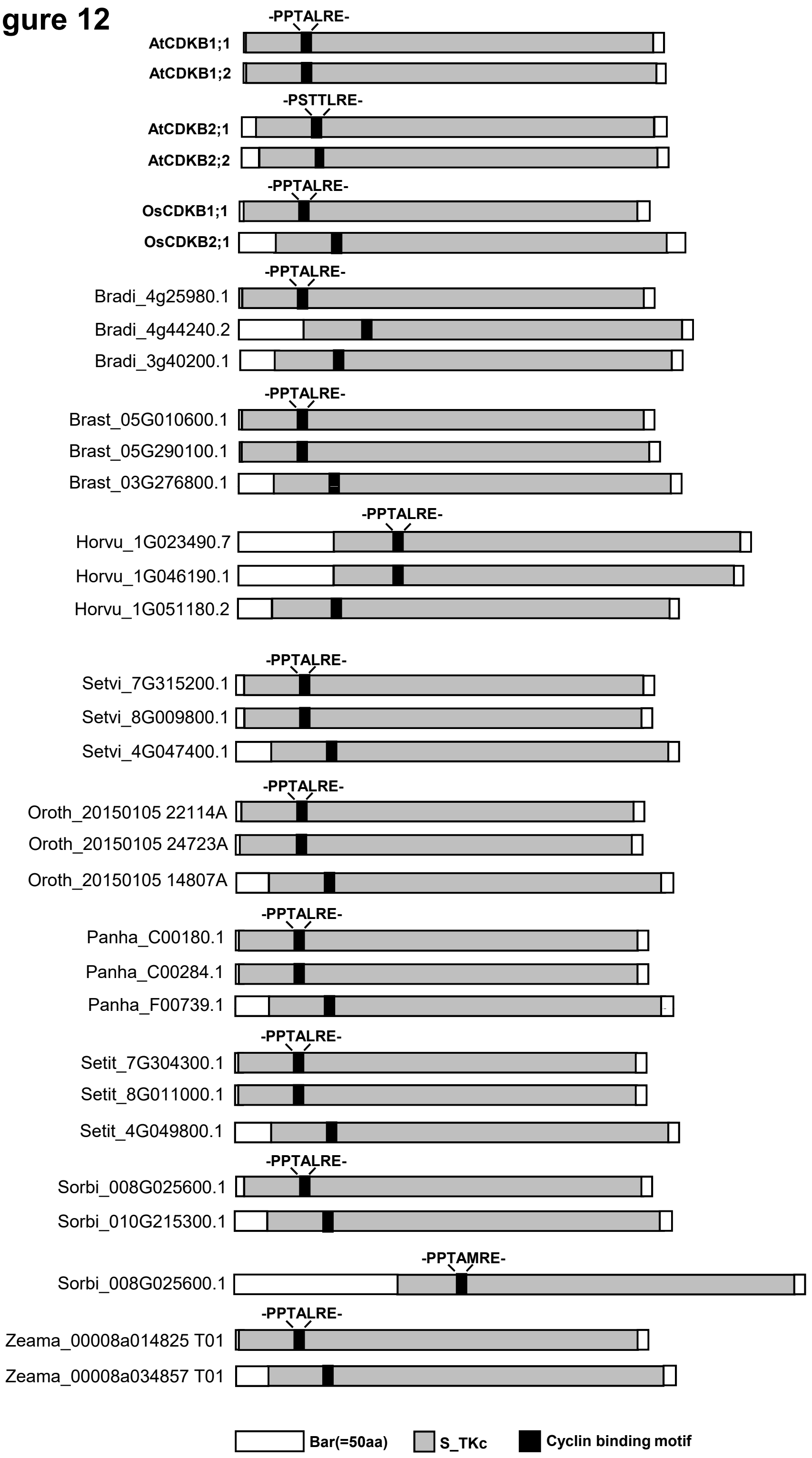

Fig. S12. CDKB1 and CDKB2 contain the same cyclin binding domain in most monocots. Schematic diagram of CDKB1 and CDKB2 of *Arabidopsis thaliana*, *Brachypodium stacei*, *Brachypodium distachyon*, *Hordeum vulgare*, *Oryza sativa*, *Oropetium thomaeum*, *Panicum hallii*, *Panicum virgatum*, *Sorghum bicolor*, *Setaria italica*, *Setaria viridis*, and *Zea mays*. The gray boxes indicate S_TKc, which is the Serine/Threonine kinase catalytic domain. The black boxes indicate the cyclin interaction domain.
